# Biotechnological Potential of Bacteria Isolated from the Sea Cucumber *Holothuria leucospilota* and *Stichopus vastus* from Lampung, Indonesia

**DOI:** 10.3390/md17110635

**Published:** 2019-11-08

**Authors:** Joko T. Wibowo, Matthias Y. Kellermann, Dennis Versluis, Masteria Y. Putra, Tutik Murniasih, Kathrin I. Mohr, Joachim Wink, Michael Engelmann, Dimas F. Praditya, Eike Steinmann, Peter J. Schupp

**Affiliations:** 1 Carl-von-Ossietzky University Oldenburg, Institute for Chemistry and Biology of the Marine Environment (ICBM), Schleusenstraße 1, D-26382 Wilhelmshaven, Germany; matthias.kellermann@uni-oldenburg.de (M.Y.K.); dversluis@hotmail.com (D.V.); 2 Research Center for Oceanography LIPI, Jl. Pasir Putih Raya 1, Pademangan, Jakarta Utara 14430, Indonesia; mast001@lipi.go.id (M.Y.P.); tuti007@lipi.go.id (T.M.); 3 Helmholtz Centre for Infection Research, Inhoffenstraße 7, 38124 Braunschweig, Germany; kathrinmohr4@gmail.com (K.I.M.); joachim.wink@helmholtz-hzi.de (J.W.); 4 TWINCORE-Centre for Experimental and Clinical Infection Research (Institute of Experimental Virology) Hannover. Feodor-Lynen-Str. 7-9, 30625 Hannover, Germany; michael.engelmann@ruhr-uni-bochum.de (M.E.); Dimas.Praditya@ruhr-uni-bochum.de (D.F.P.); Eike.Steinmann@ruhr-uni-bochum.de (E.S.); 5 Department of Molecular and Medical Virology, Ruhr-University Bochum, 44801 Bochum, Germany; 6 Research Center for Biotechnology, Indonesian Institute of Science, Jl. Raya Bogor KM 46, 16911 Cibinong, Indonesia; 7 Helmholtz Institute for Functional Marine Biodiversity at the University of Oldenburg (HIFMB), Ammerländer Heerstrasse 231, D-26129 Oldenburg, Germany

**Keywords:** marine bacteria, sea cucumber, anti-infective marine derived compounds, de-replication, mass spectrometry

## Abstract

In order to minimize re-discovery of already known anti-infective compounds, we focused our screening approach on understudied, almost untapped marine environments including marine invertebrates and their associated bacteria. Therefore, two sea cucumber species, *Holothuria leucospilota* and *Stichopus vastus*, were collected from Lampung (Indonesia), and 127 bacterial strains were identified by partial 16S rRNA-gene sequencing analysis and compared with the NCBI database. In addition, the overall bacterial diversity from tissue samples of the sea cucumbers *H. leucospilota* and *S. vastus* was analyzed using the cultivation-independent Illumina MiSEQ analysis. Selected bacterial isolates were grown to high densities and the extracted biomass was tested against a selection of bacteria and fungi as well as the hepatitis C virus (HCV). Identification of putative bioactive bacterial-derived compounds were performed by analyzing the accurate mass of the precursor/parent ions (MS^1^) as well as product/daughter ions (MS^2^) using high resolution mass spectrometry (HRMS) analysis of all active fractions. With this attempt we were able to identify 23 putatively known and two previously unidentified precursor ions. Moreover, through 16S rRNA-gene sequencing we were able to identify putatively novel bacterial species from the phyla Actinobacteria, Proteobacteria and also Firmicutes. Our findings suggest that sea cucumbers like *H. leucospilota* and *S. vastus* are promising sources for the isolation of novel bacterial species that produce compounds with potentially high biotechnological potential.

## 1. Introduction

Many marine invertebrates, particularly sessile or slow-moving organisms, are a rich source of valuable bioactive metabolites. Among marine invertebrates, sea cucumbers, or holothurians, have been utilized as food and folk medicines by Asia and Middle East communities [1]. Asian people, especially Chinese believe that consuming holothurians may treat a variety of impediments and illnesses such as weakness, impotence, debility of the aged, constipation due to intestinal dryness, and frequent urination [2]. As a consequence, these reported beneficial effects lead to the high demand for holothurians in Chinese markets.

Among holothurians, *Stichopus vastus* and *Holothuria leucospilota* were reported to have medicinal application. *S. vastus* is well-known for its wound healing activities which were proven by pre-clinical test in rats [3]. In addition, the integument tissue is rich in collagen and can be used as a functional ingredient in nutraceuticals, cosmetics and food products [4]. Furthermore, *S. vastus* contains novel bioactive peptides which inhibit angiotensin I converting enzyme (ACE) and also possesses radical scavenging activities [5]. *H. leucospilota* is widespread throughout the Red Sea, Persian Gulf and the entire Indo-Pacific. Its main habitat are shallow areas, such as reef flats, shallow costal lagoons, and seagrass beds. In some areas such as the Federated States of Micronesia (FSM), Marshall Islands, Kiribati, Samoa, Tonga, Cook Islands, Papua New Guinea (PNG), Solomon Islands and Fiji, people consume holothurian´s gonad as food delicacies and as additional protein diets [6]. *H. leucospilota* has shown antioxidant effects as well as anticancer activities against HeLa, human lung carcinoma (A549) and skin melanoma (B16F10) cells [7,8,9,10]. Several bioactive compounds have been isolated from it such as leucospilotaside A to C, echinoside B, holothurin A, holothurin B, and holothurin B2 [11]. Organic extracts of body wall, gonad and intestine of *H. leucospilota* exhibited bacteriostatic rather than bactericidal activity against Gram-positive *Bacillus subtilis* and *Staphylococcus aureus* [12]. This bacteriostatic effect from the organic extracts was confirmed against the Gram-negative bacteria like *Escherichia coli*, *Salmonella typhi*, and *Pseudomonas aeruginosa* and against the Gram-positive *S. aureus* and the filamentous fungi *Aspergillus niger*, *A. fumigatus*, *A. flavus* and *A. brasilensis* [13].

Numerous natural products from marine invertebrates show striking structural similarities to those of microbial origins, suggesting that microorganisms are at least involved in the biosynthesis of the targeted bioactive compound or they even represent the sole producer of the respective metabolites [14,15]. Those findings supported efforts to isolate invertebrate associated bacteria as the real producer of the bioactive compounds to overcome the supply problem as harvest from the wild is not sustainable for most bioactive marine invertebrates [16]. 

Bioprospecting for bioactive marine bacteria recognizes the noticeable capacity of marine bacteria as a source of new natural products which can be utilized to overcome the antimicrobial resistance crisis [17]. (Multi-)drug-resistant bacteria are becoming the global challenge leading to the strong demand for new antibiotics, either in chemical structures or mode of actions [18]. In addition, spreading of slow progressing but deadly virus, such as Hepatitis C (HCV) menace human population particularly in developing countries. Therefore, the detection and development of new anti-infective drugs is urgently needed [19,20].

There has been an increasing number of publications focusing on the isolation of invertebrate associated bacteria for the discovery on new bioactive compounds, with sponges being the invertebrate phylum that has received most attention for isolation of the associated microbiome [21,22,23,24,25,26,27,28]. However, holothurians likely present another very interesting target for the isolation of bioactive bacteria. Since they are being (a) used in traditional Chinese medicine and (b) exposed to, ingesting and reworking marine sediments, which have been shown to be a promising source for the isolation of bioactive bacteria. Even more so, if Actinobacteria, which are prolific producers of bioactive compounds, are the target bacteria phylum, since Actinobacteria have been isolated repeatedly from terrestrial soil and marine sediments. Thus, sediment bioturbating holothurians with their associated microbiome should be a promising target to isolate novel Actinobacteria. A recent publication by Gao, et al. (2014) showed that Actinobactria were enriched in the gut of four deep-water holothurian, accentuating that also shallow water holothurians could be a promising target for the isolation of bioactive Actinobacteria [29].

In this study, we reported the potential of bioprospecting underexplored marine associated bacteria derived from the sea cucumbers *H. leucospilota* and *S. vastus*.

## 2. Results

### 2.1. Bacterial Isolation, Taxonomic Identification, and Antimicrobial Assay

In this study, a total of 275 bacterial colonies were isolated in Indonesia from the internal and external parts of the two sea cucumbers, *Holothuria leucospilota* (HL) and *Stichopus vastus* (SV). Back in Germany, bacterial colonies were re-grown on marine agar (MA) resulting in 127 different strains based on 16S rRNA gene sequence analyses (Table 1). A detailed compilation of all isolated bacterial strains from HL and SV are shown in Table S1. Our results of the identified phyla are in line with previous studies that attempted to isolate cultivable bacteria from marine macroorganisms and environments [30,31,32]. However, as determined by next generation sequencing studies, the cultivable bacteria still only represent about 1% of the estimated microbial diversity [33].

From the identification result, some Actinobacteria from *H. leucospilota* (HL 108, HL 111, HL 255, HL 66 and HL 268) show less than 98% similarity to the next type strains when compared to those in the NCBI database (http://www.ncbi.nlm.nih.gov/; using the Basic Local Alignment Search Tool (BLAST)) and thus are representatives of a putative new bacterial species. HL 108 is related to *Glutamicibacter nicotianae* (96.38% sequence similarity), HL 111 to *Nocardioides exalbidus* (97.96% sequence similarity), HL 255 to *Kytococcus sedentarius* (97.58% sequence similarity)*,* and the others to *Kocuria palustris* (97.64% and 97.45% sequence similarity), respectively (Table S1). 

Two Actinobacteria from *S. vastus*, SV 16 and SV 203, showed less than 98% similarity to the next type strain. These bacteria are related to *Serinicoccus profundi* (97.91% sequence similarity) and *Mariniluteicoccus endophyticus* (96.26% sequence similarity), respectively (Table S1). In addition, an Actinobacteria from *S.vastus* showed less than 95% similarity the next type strains. Isolated from the external part of *S. vastus*, SV 17 is putatively a member of a new genus of the Propionibacteriaceae (93.3% sequence similarity with *Pseudopropionibacterium rubrum*). The phylogenetic position, based on 16S rRNA-gene analyses, of selected isolated Actinobacteria is presented in Figure S1.

A total of 33 strains of Firmicutes that related to the genera *Staphylococcus* and *Bacillus* could be isolated from both, *H. leucospilota* and *S. vastus* (cf. Figure S2). Bacterium HL 79 showed only 92.88% similarity to the next type strain *Bacillus sonorensis*, and thus probably represents a new genus (Table 2). Isolates that had more than 99% sequence similarity with *Staphylococcus cohnii* subsp. *urealyticus* were found in all samples.

We identified 30 strains belonging to 9 different genera of Proteobacteria (Phylogeny tree see Figure S3). The genera *Vibrio* and *Paracoccus* were found in both *H. leucospilota* and *S. vastus*. *Vibrio alginolyticus* was isolated from both sea cucumbers (Table 2). Both *Vibrio alginolyticus* and *Vibrio harveyi* have caused diseases in aquatic animals including sea cucumbers [34,35]. Pathogenicity of the genus *Vibrio* is not only caused by suitable conditions (i.e., temperature, low host immunity and nutrition) but also by the presence of the vibriolysin-like protease [36].

Analyses of sequences resulted in several Proteobacteria showing less than 98% similarity to the next type strains: HL 125 showed 96.23% sequence similarities to *Vibrio harveyi* and HL 28 showed 97.33% similarity to *Paracoccus koreensis*. These bacteria probably represent new species. Bacterium SV155 is putatively a member of a new genus of the Rhodobacteraceae which showed closest relationship to the genus *Paracoccus* (93.08% sequence similarity with *Paracoccus beibuensis*, Table 2).

We tested all 127 bacterial strains with the agar plug diffusion assay against environmental bacteria in a preliminary screening. Subsequently the 69 active strains were further cultured and extracted for additional bioassays. There were 19 bacterial strains that active in the preliminary test from 39 bacterial strain from internal part of *H. leucospilota* extracted and further assayed against microorganisms. About 47.4% (9 out of 19) of the bacterial extracts from the internal part of the *H. leucospilota* showed activity against Gram-positive *Bacillus subtilis* and 15.8% (3 out of 19) were active against *Staphylococcus aureus*. Strain HL 55, identified as *Kocuria flava* (99.21% sequence similarity), displayed potent activity against the two Gram-positives strains and additional activity against the Gram-negative *E. coli* (Table 2). Only one strain from the internal parts of the *H. leucospilota* showed activity against filamentous fungi *M. hiemalis* (5.3%, 1 out of 19). However, fungal activity was markedly higher against filamentous fungi *M. hiemalis* and *R. glutinis* when bacterial extracts from the external parts of *H. leucospilota* were tested (22.2%, 6 out of 27 tested).”

Antimicrobial testing on 27 bacterial strains from the external part of the *H. leucospilota* which active in the preliminary screening showed as much as 44.4% (12 out of 27) were active against *B. subtilis*, 51.9% were active against *S. aureus*, 3.7% were active against *M. smegmatis*, 7.4% active against *M. hiemalis*, and 25.9% active against *R. glutinis.* Strains with the highest activity were HL 22, HL 63, HL 67, HL 111, which related to *Vibrio alginolyticus, Bacillus safensis, Staphylococcus cohnii* subsp. *urealyticus*, and *Nocardioides* sp., respectively (Table 2). The observed antimicrobial activities are in line with previous studies [37,38,39], but bioactivities on *S. cohnii* have not been reported so far.

From 23 different bacterial strains that showed already activity in the preliminary screening, bioactive compound producing strains isolated from *S. vastus* were identified as *Streptomyces cavourensis* (SV 21), *Bacillus safensis* (SV 147), and a putatively new genus of the Rhodobacteraceae which closely related to genus *Paracoccus* (SV 155, Table 2). *Streptomyces cavourensis* has been reported to strongly inhibit plant pathogenic fungi [40]. The antimicrobial activity of *Paracoccus* spp. has been recorded against *Salmonella* sp., *Proteus* sp., and MRSA [41]. In addition, recent studies reported algicidal activities of *Paracoccus* sp. against harmful algal blooms of *Prorocentrum donghaiense* [42].

### 2.2. Illumina MiSEQ Analysis from the Tissue of Sea Cucumber

**Figure 1:**
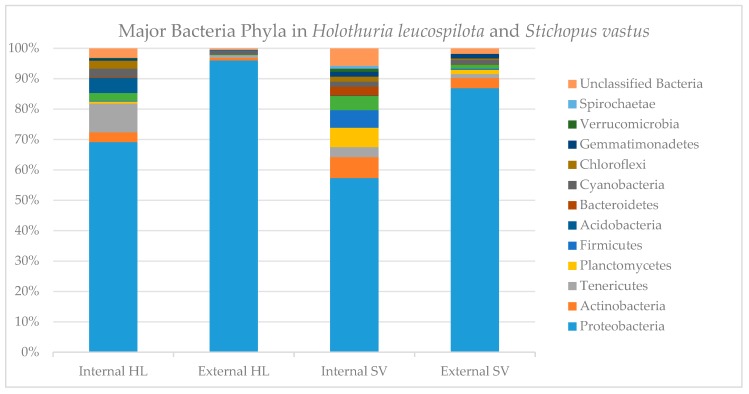
Graph shows the relative abundance of OTU on a Phylum level (HL = *Holothuria leucospilota*, SV = *Stichopus vastus*).

In total, there were 12 major phyla detected from the external and internal tissue samples from the sea cucumber *H. leucospilota* and *S. vastus* (Figure 1). Proteobacteria were the most abundant one. This result is comparable with a previous study on the bacterial communities from the gut content and ambient sediment from *Stichopus japonicus* [29]. Previous study on microbial diversity of the coelomic fluid of *H. leucospilota* found at least five bacterial genera from two phyla such as *Bacillus* and *Exiguobacterium* from phylum Firmicutes. Meanwhile *Pseudomonas, Stenotrophomonas* and *Vibrio* from phylum Proteobacteria [43]. 

Other phyla such as Firmicutes, Bacteroidetes and Actinobacteria were also detected and thus resembles the results from the cultivable approach. Interestingly, relative abundance of Proteobacteria was lower in the internal parts compared to the external parts of both species, while relative abundance of other phyla increased. For example, relative abundance of Actinobacteria was 3.6 and 9.6 times higher in the internal parts compared to the external parts of *S. vastus* and *H. leucospilota*, respectively. 

### 2.3. Testing the Effect of Bacterial Extracts on the Infectivity of Hepatitis C Virus (HCV)

**Figure 2:**
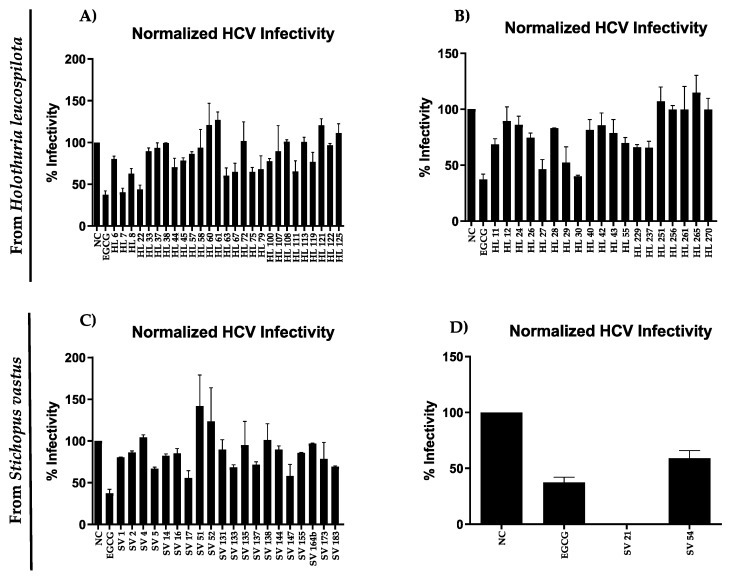
(**A**) and (**C**) Inhibition of HCV infectivity of extracts derived from bacterial isolates of the external sea cucumber parts; (**B**) and (**D**) Inhibition of HCV infectivity of extracts derived from bacterial isolates of the internal sea cucumber parts. NC-negative control, epigallocatechin gallate (EGCG)-positive control. Viability assay results are given in the supplementary section (Figure S4).

This screening identified four bacterial extracts from *H. leucospilota*, which inhibited HCV infectivity by more than 50% (Figure 2), while all showed no cytotoxicity on the liver cells compared to negative control (Figure S4). The green tea molecule epigallocatechin gallate (EGCG) was used as positive control [44]. Both bacterial extracts HL 7 from the internal part and HL 30 from the external part of the *H. leucospilota* showed the strongest inhibition of HCV infectivity. They also showed low activity against *B. subtilis* (Table 2). Partial identification with Sanger sequencing of HL 7 showed 100% similarity to *Kocuria palustris*, while HL 30 showed 99.88% similarity with *Kytococcus sedentarius*. 

On the other hand, only one bacterial extract from both the internal and external part of *S. vastus* showed an inhibitory effect of more than 50% inhibition against HCV. Bacterial extract *S. cavourensis* SV 21 resulted in a very strong inhibition of HCV infectivity, but also displayed toxicity towards the target cells. These effects may be caused by the high concentration of the active compound in the extract. 

Bacterium SV 17 was putatively a member of a new genus of the Propionibacteriaceae (93.3% sequence similarity with *Pseudopropionibacterium rubrum*). Both bacteria SV 17 and SV 147 (100% sequence similarity with *Bacillus safensis*) were isolated from the external part of *S. vastus* and both their bacterial extracts revealed almost 50% inhibition of HCV infectivity (cf. Figure 2C). *Bacillus* sp. has been reported to have bioactivities against HCV [45], but this could be the first report of antiviral activity against HCV infectivity by a bacterium which has closest sequence similarity to genus *Pseudopropionibacterium*.

### 2.4. Identification of Putative Compounds from Bioactive Fractions

All bacterial extracts that showed high activities against any tested pathogen were fractionated further in order to isolate and potentially identify the responsible bioactive compounds. Preliminary compound identification was done by comparing exact mass of the precursor/parent ions (MS^1^) of the active fractions with known databases (i.e., MarinLit, Dictionary of Natural Products (DNP), METLIN, and Global Natural Product Social Molecular Networking (GNPS)). Furthermore, we analyzed the product/daughter ions (MS^2^) of the respective peaks by comparing the most prominent MS^2^ spectra with available databases (METLIN, GNPS) and/or literature. A summary of this approach is provided in Table 3. 

The aim of the chemical analysis, using MS^1^ and MS^2^ spectral data, was to determine compounds in the bioactive fractions by comparing mass spectral data with other databases and/or literature. Dereplication that only considers MS^1^ to determine putative compounds has been somewhat unreliable due to isobaric compounds. Fragmentation spectra (MS^2^ spectral data) have become necessary in order to support results from the MS^1^ analysis [46]. Such comprehensive information is crucial to prioritize samples for further isolation of the potential novel anti-infective compounds.

In this study, 25 precursors have been found from eight different bacterial extracts (Table 3). From these 25 targets only 20% (5 out of 25) were unknown based on the comparison of the exact mass (±0.005 Da) from the precursor ions (MS^1^) with databases (MarinLit, DNP, METLIN and GNPS). Based on the MS^2^ spectra, as much as 28% (7 out of 25) could not be matched to a known candidate compounds or having a low match with known compounds by either showing a low cosine score or low numbers of shared peaks. Only 8% (2 out of 25) were unidentifiable in both, MS^1^ and MS^2^ analysis. 

The comparison of the exact masses of the precursor ions (±0.005 Da) to compounds in the databases often resulted in similarities with multiple compounds isolated from various organisms (cf Table S2). In these cases, we also compared the literature MS^2^ data of the compounds with our MS^2^ sample data. 

The analysis of the active fraction of *S.cavourensis* SV 21 resulted in the detection of five precursor ions (Table 3). Results of the search in the MS^1^ databases can be found in Table S2. Precursor ion of *m*/*z* 458.181 [M + H]^+^ matched with the previously identified compound medermycin (457.173 [M]) from *Streptomyces* sp. This result was supported by the results of the MS^2^ experiments and comparison of the obtained mass data with the GNPS database (cosine score 0.74, by only 10 shared peaks). Intriguingly, medermycin’s precursor in the GNPS database was *m*/*z* 457.17 with adduct ion [M + H]^+^. A difference of 1.01 with the precursor in our sample and also with the exact mass in the MS^1^ databases. 

One of the closest matched compounds for the precursor ion *m*/*z* 490.207 was the antibiotic OA 6129E (489.2145 [M]), which had been originally isolated from *Streptomyces* sp. OA-6129 [47]. The MS^2^ spectra of the compound was not mentioned in those articles. However, comparison of the MS^2^ spectra 490.207 [M + H]^+^ with the GNPS Database resulted in low similarity to candesartan (precursor ion of *m*/*z* 441.17, cosine score of 0.61, and only 7 shared peaks). Even though the cosine score were high, the shared peaks were low. Besides, the putative compound was not reported from *Streptomyces*. Thus, further isolation, activity testing and compound identification is needed.

**Figure 3:**
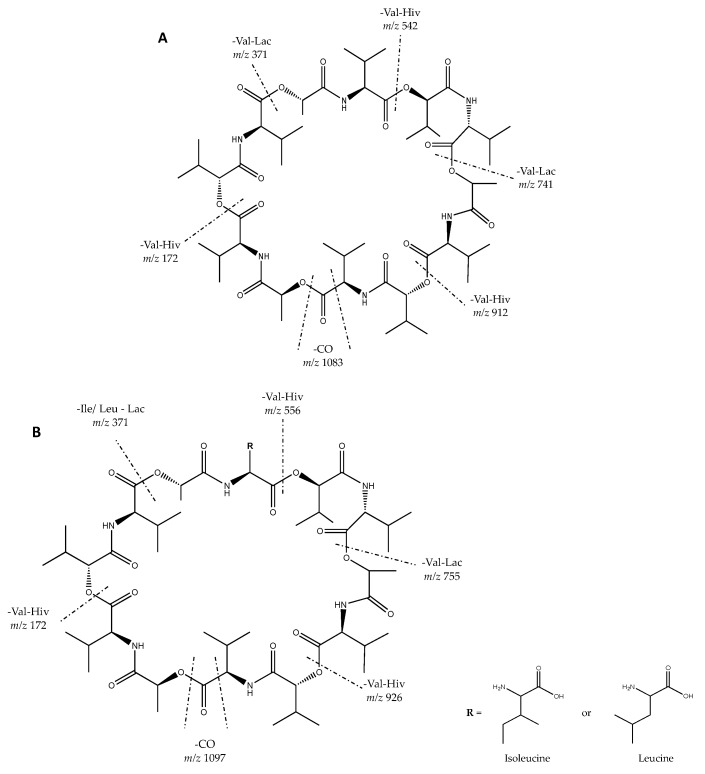
Logic MS^2^ interpretation of putative structure of: (**A**) valinomycin (*m*/*z* 1128.66 [M + NH_4_]^+^) and (**B**) its derivate (*m*/*z* 1142.67 [M + NH_4_]^+^). The difference between A and B is 14 Da. It might have from the substitution of valine with either isoleucine or leucine. Figure was adapted from [48].

An interesting compound was detected in the active fraction from *S. cavourensis* SV 21 with a precursor ion of *m*/*z* 1142.67 [M + NH_4_]^+^. Based on the MS^1^ analysis, the precursor had not been reported in the databases yet. However, the MS^2^ data of the compound have been reported in [48]. It showed a difference of 14.01 Da with valinomycin (precursor *m*/*z* 1128.66 [M + NH_4_]^+^, cf. Figure S5). The MS^2^ spectra of the precursor ion of *m*/*z* 1142.67 [M + NH_4_]^+^ also showed high similarity to valinomycin in the GNPS library with a cosine score of 0.82 and 46 shared peaks (Figure S6). It indicated that the unidentified compound was putatively a valinomycin derivate with a molecular mass difference of 14.01 Da with valinomycin. The fragmentation pattern of valinomycin and its derivate (Figure 3) showed a difference in the substitution of valine with either isoleucine or leucine. All of the mentioned fragments in Figure 3 can be found in the MS^2^ spectra in Figures S5 and S6. Further isolation and identification are needed for confirmation of the chemical structure. 

Precursor *m*/*z* 663.454 [M + H]^+^ in the active fraction from *S. cavourensis* SV 21 showed matches with multiple compounds based on the MS^1^ analysis. Further analysis using the MS^2^ GNPS database resulted in the match with sarmentoside B with a cosine score of 0.76 and 19 shared peaks. Interestingly, sarmentoside B (*m*/*z* 665.317 [M + H]^+^) is a glycoside from the plant *Strophanthus sarmentosus* [49]. It has not been reported from bacteria yet. However, further isolation and identification of the compound is needed for the confirmation. 

**Figure 4:**
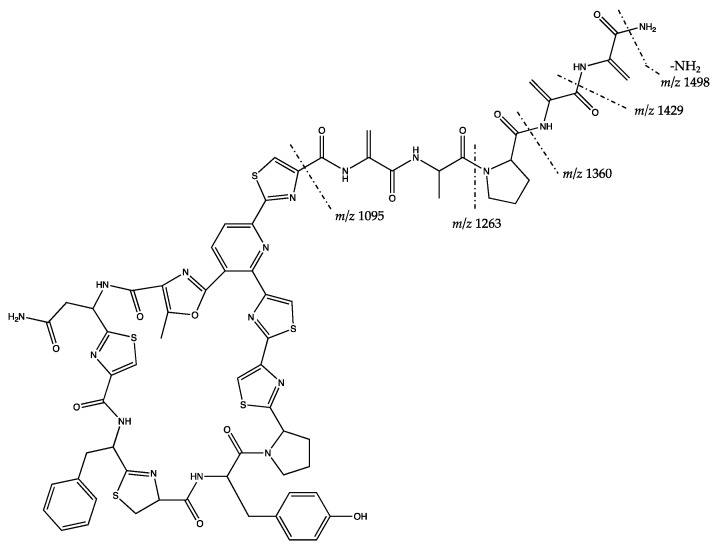
MS^2^ interpretation of putative structure of kocurin (*m*/*z* 1515.373 [M + H]^+^). Figure was adapted from [50].

The two putative bioactive precursor ions, 1140.219 [M + H]^+^ and 1515.373 [M + H]^+^, were identified from the fraction of *Kocuria flava* HL 55. The detected precursor ion 1140.219 [M + H]^+^ is to this point unidentified and has neither been reported by MS^1^ nor by MS^2^ (cf. Figure S7). Based on the MS^1^ and MS^2^ spectra from the literature, the precursor ion 1515.373 was identified as kocurin [50]. The MS^2^ logic of the kocurin fragmentiation showed similar fragments with [50], except for the *m*/*z* 1095 (Figure 4). All of the mentioned fragments can be seen in the MS^2^ spectra in Figure S8. 

**Figure 5:**
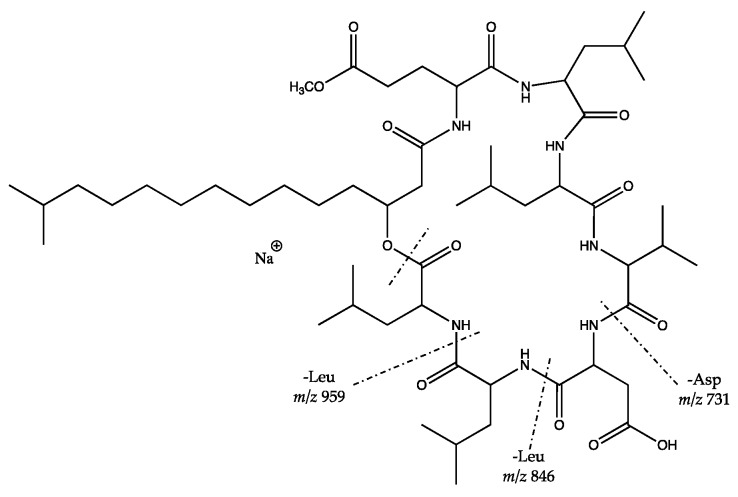
MS^2^ interpretation of putative structure of surfactin (1072.686 [M + Na]^+^). Figure was adapted from [51].

As much as 11 precursors were detected in the active fraction from *Bacillus safensis* HL 63 and *Staphylococcus cohnii* subsp. *urealyticus* HL 67. The precursors *m*/*z* 1070.643 [M + H]^+^, 1076.629 [M + Na]^+^, 1068.661 [M + H]^+^, 1022.674 [M + H]^+^, 1058.671 [M + Na]^+^, 1072.686 [M + Na]^+^, 1096.692 [M + H]^+^, 1086.702 [M + Na]^+^, 1102.616 [M + H]^+^, and 1100.717 [M + Na]^+^ were identified as putative-surfactins in MASST GNPS (exemplified in Figures S9 and S10). The logic MS^2^ fragments were exemplified by *m*/*z* 1072.686 [M + Na]^+^ (Figure 5) which were similar to surfactins reported in [51], except for the fragments below *m*/*z* 731. This finding confirmed the result of MS^1^ databases search for precursors *m*/*z* 1022.674 [M + H]^+^, 1058.671 [M + Na]^+^, and 1072.686 [M + Na]^+^ (cf. Table S2).

While a precursor *m*/*z* 875.534 [M + Na]^+^ (Figure S11) in the active fraction from *Bacillus safensis* HL 63 and *Staphylococcus cohnii* subsp. *urealyticus* HL 67 had not been reported by MS^1^ nor by MS^2^.

The two precursor ions *m*/*z* 347.212 [M + H]^+^ and *m*/*z* 395.213 [M + H]^+^ were identified from the active fraction of *Staphylococcus edaphicus* HL 75. However, both precursor ions had similar exact masses with multiple compounds found in the databases. The MS^2^ analysis resulted in low similarity to (-)-pipoxide (precursor *m*/*z* 367.12 [M + H]^+^) and to 4-acetyloxy-8-(3-oxo-2-pent-2-enylcyclopenten-1-yl) octanoic acid (precursor *m*/*z* 349.2 [M − H]^-^), respectively. Those compounds had not been reported from bacteria. Therefore, further isolation for identification of the compounds for respective precursors are needed.

There were five detected precursor ions that were identified in the active fraction of *Bacillus safensis* SV 147, *Paracoccus beibuensis* SV 155 as well as *Nocardioides exalbidus* HL 111. 

**Figure 6:**
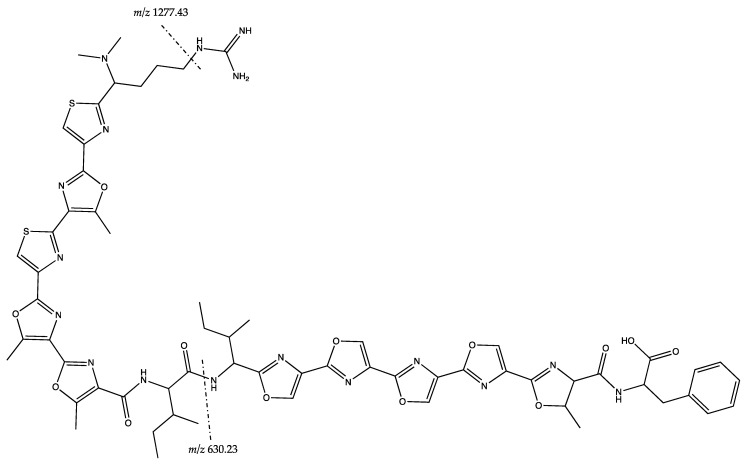
MS^2^ interpretation of putative structure of plantazolicin A *m*/*z* 1336.478 [M + H]^+^. Figure was adapted from [52].

The precursor *m*/*z* 1336.478 [M + H]^+^ had the closest exact mass to plantazolicin A, a compound isolated from *Bacillus* sp. [52]. MS^2^ analysis with MS^2^ spectra from [52] gave shared product ion peaks with *m*/*z* 455.059; 523.122; 630.230; 679.259; as well as 1277.425 (MS spectra of the sample is shown in Figure S12). The MS^2^ spectra in Figure 6 showed the product ions of *m*/*z* 1277.43 and 630.23 from plantazolicin A. Therefore, the precursor of *m*/*z* 1336.478 [M + H]^+^ was putatively assigned as plantazolicin A. Further isolation and identification of the compound is needed for confirmation.

The precursor ions *m*/*z* 1044.657 [M + Na]^+^, 1058.671 [M + Na]^+^, 1050.705 [M + H]^+^ and 1086.703 [M + Na]^+^ have similar fragment profiles with *m*/*z* 1072.686 [M + Na]^+^ as mentioned earlier. Thus, they were identified as putative-surfactins. These results indicate that bacteria from different taxa are able to produce the same compounds. Further analyses are needed to confirm the chemical composition of these bacteria and whether these bacteria have the same bioactive gene clusters (BGCs). 

## 3. Discussion

A first step in the drug discovery process is the identification of novel bioactive compounds or known compounds with newly identified bioactivities. One approach has been high throughput screening of synthetic and/or natural products libraries, which is not really possible in University settings due to lack of financial and human resources. Another approach is to focus on drug discovery from unusual environments and unusual biological sources. As many of the terrestrial environments have been investigated in detail for decades, screening of terrestrial organisms and plants is increasingly yielding known compounds rather than novel compounds and new drug leads. Likewise, antibiotic screens from soil derived microorganisms often resulted in the re-discovery of already known antibiotic compounds [53]. To circumvent re-discovery, untapped sources such as various marine ecosystems (e.g., coral reefs, twilight zone habitats) are getting in the focus of researchers [54]. For example, marine bacteria such as Actinobacteria and Bacilli derived from marine sediments have been proven to be a valuable source of new antibiotics [55].

Bacteria associated with marine invertebrates can be a promising source of new antimicrobial compounds, as confirmed by the identification of potent antimicrobial extracts from the sea cucumber *S. vastus* and *H. leucospilota* associated bacteria. The isolated Actinobacteria produced interesting bioactive metabolites, which exhibited activity against HCV, bacteria, and fungi. In addition, the potentially novel Actinobacteria genera and species displayed anti HCV properties, requiring further research on these bacteria to identify the new anti-infective compounds. It is known that secondary metabolite producing bacteria synthesize different metabolites under certain environmental conditions (i.e., temperature, salinity, O_2_ stress, different media composition) or when co-cultured with other microorganisms. Such an approach could be a promising strategy for the discovery of new anti-infectant compounds with the new bacteria genera and species.

The main underlying reasons on performing a screening approach for new anti-infective compounds from sea cucumber derived bacteria was the biotechnological potential and ecological roles of the hosts [1,56]. There are several reasons why sea cucumbers are a promising source of novel bacteria and potentially bioactive compounds: (1) Sea cucumber have an important ecological role in the marine environment through bioturbation of the sediment, thereby extracting and removing organic material, microalgae and bacteria from the sediment and defecating sediment with a lower organic content [57]. During this process they likely enrich certain bacteria in their gut microbiome [29]. (2) Due to their slow movement and soft to leathery body walls, sea cucumbers rely mainly on potent chemistry for their defense against predators such as fishes. Echinoderms in general are known to have a diverse metabolome that can be highly affected by their surrounding environment and their diet [58]. However, up to this point, it is not known whether the bioactive compounds are produced by the host or the associated microorganisms. 

The role of bacteria for the sea cucumber, especially the producers of bioactive compound, remains unclear. Associated Actinobacteria could be an ecological advantage by providing the host with bioactive compounds for i.e., protection against infection by pathogenic bacteria or protection against predators [59]. The function of *Staphylococcus* bacteria in the sea cucumbers is still unknown, but a study suggested that the orange color in the respiratory track of *H. leucospilota* may be an result of the pigment-producing strain *Staphylococcus klosii* [60]. 

Compared to the overall sea cucumber microbiome of *S. vastus* and *H. leucospilota,* Actinobacteria are only represented by a small number of associated bacteria. The majority of the associated bacteria belong to the phylum Proteobacteria (Figure 1). In this study, some identified Proteobacteria showed antimicrobial activities and also produced putatively novel compounds, and thus emphasizing the need to follow up the identified leads for bioactive compounds. In addition, we also isolated putatively new species of bacteria which belonged to the phyla Proteobacteria and Actinobacteria, representing further opportunities for the discovery of novel bioactive metabolites.

Bioassays of the bacterial extracts identified eight promising bacterial strains that were derived mostly from Actinobacteria and Firmicutes. For example, *Streptomyces cavourensis* SV 21 showed strong activity against bacteria and HCV (Table 2, Figure 2). Analysis of the active fraction of *Streptomyces cavourensis* SV 21 showed five precursors. The precursor ion of the largest peak was identified as valinomycin. Valinomycin, a cyclodepsipeptide, holds a potent antibiotic activity that had been previously recovered from various soil-derived Actinomycetes, such as *S. fulvissimus*, *S. roseochromogenes* and *S. griseus* var. *flexipartum* [61] as well as from marine *Streptomyces* species that were associated with the sponge *Axinella polypoides* and *Aplysina aerophoba* [62]. Another interesting bioactivity of valinomycin was its potency against the causative agent of the world’s first pandemic in the 21^st^ century; the SARS-CoV virus. Unfortunately, valinomycin also showed enhanced cytotoxicity that prevented the drug to enter the clinical phase [63,64]. The other precursor ions (cf. Table 3) were either unidentified compounds or could only be partially identified. Therefore, in order to define the additional active compounds and to determine their structure, further isolation and identification are needed.

We also found an unidentified compound from the active fraction of *Kocuria flava* HL 55 with the precursor ion of *m*/*z* 1140.219 [M + H]^+^. It eluted in the UPLC-HRMS (Waters Synapt G2-Si, Milford, MA, USA) chromatogram close to the precursor ion *m*/*z* 1515.373 [M + H]^+^ which was identified as kocurin. Kocurin, isolated from *Kocuria palustris,* was previously described as potent antibiotic compound against methicillin-resistant *Staphylococcus aureus* (MRSA) [50]. Another study of a bacterium with 96% sequence similarity to *Kocuria flava* S43 was able to inhibit bacteria causing coral disease, so called yellow blotch [65]. Detection of kocurin in extracts from this study indicated that it seems to be a common metabolite produced by bacteria of the genus *Kocuria*.

UPLC-HRMS analysis of the active fraction from *Bacillus safensis* HL 63 and *Staphylococcus cohnii* subsp. *urealyticus* HL 67, revealed an unidentified precursor ion with the mass of *m*/*z* 875.534 [M + Na]^+^.

In this study, the two precursor ions of *m*/*z* 1140.219 [M + H]^+^ (from *Kocuria flava* HL 55) & 875.534 [M + Na]^+^ (from *Bacillus safensis* HL 63 and *Staphylococcus cohnii* subsp. *urealyticus* HL 67) could not be identified in both, MS^1^ and MS^2^ databases, and thus these two remain, to the best of our knowledge, as unidentified and might be putative novel bioactive compounds. However, one should keep in mind that identified precursor ions in either MS^1^ or MS^2^ compound databases might still be false positive, if they contained only a low number of matched peaks of their product/daughter ions. Precursor annotation as false positive in MS^2^ databases has been reported to be in linear correlation with true positive precursor annotation [66]. It means, as more compounds or libraries are added to MS^2^ databases, the probability of a false positive analysis of the target compounds becomes higher. Orthogonal analysis of the compounds (cf. Table 3.) that are responsible for the putatively new or partially identified precursors need to be done in order to find the actual structure of the compounds.

We isolated and tested some bacteria from the same species i.e, *Kocuria palustris* and *Kytococcus sedentarius*, but not all of them had the same bioactivities. This might be caused by the different compounds produced, even within the same species. A study by [67] showed that bacterial strains which are identical based on their 16S rRNA gene sequence similarity can actually produce different secondary metabolites, as the overall genome of the strains could be still somewhat different and therefore encode for different metabolites.

Overall, this study confirmed that the use of understudied marine invertebrates such as sea cucumbers is a promising approach for the isolation of novel bacteria strains and identification of compounds in bioactive fractions. 

## 4. Materials and Methods 

### 4.1. Isolation of Bacteria

An individual of both *Holothuria leucospilota* and *Stichopus vastus* were collected in Sari Ringgung, Lampung, Indonesia (coordinates: S 05°33.706’ E 105°16.220’) on the 19 April 2016. Sea cucumbers were kept cold on ice until the bacterial isolation was carried out. 

Skin (external) and intestine (internal) parts of the sea cucumbers were used for bacterial isolation. Several media were prepared including Marine Agar 100% (MA, made from Marine Broth (MB, Carl Roth, Karlsruhe, Germany) according to manufacturer’s instruction with addition of 9 g/L agar (Agar-agar Bacteriological, Carl Roth, Karlsruhe, Germany)), Marine Agar 10% (MA2, was made by a 10-fold dilution of MA with distilled water) and M1 media (1.8% agar, 1% starch, 0.4% yeast extract, 0.2% peptone and filtered seawater). We choose the rich nutrient media MA and M1 for the isolation as it was used in the previous publications [30,31,68]. While the lower nutrient MA2 media was provided to allow slow growing bacteria more time to form colonies before the agar plates were eventually covered by fast growing bacteria like in the MA media. We swabbed with sterile cotton buds the surface of sea cucumbers after they had been washed with sterile sea water to isolate the associated bacteria. Swaps were streaked onto agar plates. In addition, a one cm piece from the outer and inner (intestine) body part was mixed with 1 mL of sterile sea water, homogenized and serially diluted to give 10×, 100×, and 1000× dilutions. The function of the dilution was to increase the percent cultivability and diversity of the bacterial isolates by reducing the competition among bacteria [69]. As much as 150 µL of each serial dilution was plated onto agar plates.

Agar plates were incubated at 28 °C for 14 days. Bacterial colonies were picked from the agar, and then re-inoculated multiple times to get pure bacterial strains. Pure cultures were transferred to 10 mL MB (Marine Broth, Carl Roth, Karlsruhe, Germany) in sterile Corning tubes. After 72 h incubation in room temperature, the glycerol stocks from each pure culture were made by mixing sterile glycerol with broth culture 3:1 in 2 mL cryo tubes and storing them in −80 °C. Before identification of the bacteria via Sanger sequencing, bacteria were visually de-replicated based on the colonies’ appearances (e.g., color, shape, optical property and size of colony) to reduce duplication. 

### 4.2. Identification of Bacteria by 16S rRNA Gene Sanger Sequencing and Construction of Phylogenetic Tree

All pure isolates were transferred onto MA. Subsequently, their identity was determined by 16S rRNA gene Sanger sequencing using universal forward primer 27F (5’-AGAGTTTGATCCTGGCTCAG-3’) and reverse primer 1492R (5’-GGTTACCTTGTTACGACTT-3’) [70]. One reaction mixture contained 10 μL 5 × GoTaq reaction buffer (Promega, Madison, WI, USA), 1 μL dNTPs (10 mM, Promega), 0.5 μL GoTaq DNA polymerase (5 u/μL, Promega), 1 μL upstream primer (10 μM), 1 μL downstream primer (10 μM), 1 μL template (briefly frozen and thawed bacterial biomass in TE Buffer) and 35.5 μL nuclease-free water (Promega). The PCR program consisted of initial denaturation at 95 °C for 5 min; 30 cycles of denaturation at 95 °C for 30 s, annealing at 52 °C for 40 s and extension at 72 °C for 90 s; and final extension at 72 °C for 7 min. Purified PCR products were checked on a 0.8% agarose gel and purified using DNAeasy Powersoil Kit (Qiagen, Venlo, The Netherlands). Purified products were sent to GATC Biotech for sequencing with primer 27F. Read ends were trimmed with DNA Baser version 3.5.4.2 (Heracle BioSoft SRL, Arges, Romania) until there were 99% good bases (quality value > 21) in a 20-base window. To identify the closest relatives, sequences were compared to those in the NCBI’s 16S ribosomal RNA gene sequences (Bacteria and Archaea) database. Sequences were deposited at NCBI database under accession number MK696422–MK696544 and MK720778–MK720780 (except for SV 155, see Table S4).

Based on the study by [71], we considered our isolates to potentially belong to novel species if they shared less than 98% sequence similarity with the closest type strain, and potentially novel genera if the sequence similarity was less than 95%. However, further phenotypic and/or genotypic characterization is required to be able to confidently assign these strains to novel taxa [72]. 

The phylogenetic tree was constructed with 16S rRNA gene bacterial sequences and the nearest type strains. From bacteria which show more than 99% similarity to each other, one representative is shown in the tree. The Neighbor-joining tree was constructed using MEGA X version 10.0.5 (Philadelphia, PA, USA). Bootstrap values greater than 50% are shown at the nodes and are based on 1000 iterations. The scale bar represents the number of base substitutions per site.

### 4.3. Preparation of 16S Amplicon Sample Library for Illumina MiSEQ

DNA was extracted from holothurian skin and gut samples immersed in 100% ethanol using the DNAeasy Powersoil Kit (Qiagen, Venlo, The Netherlands). Prior to DNA isolation, cell material was spun down and the supernatant removed. For each sample, a barcoded 16S rRNA gene PCR was performed with primers amplifying a 292 bp fragment in the V4 region as previously described [73]. The composite forward primer consisted of the Illumina 5’ adapter, a 8-nt barcode, a 10-nt pad sequence, a 2-nt linker and the 515F-Y 16S rRNA gene-specific primer, whereas the composite reverse primer consisted of the Illumina 3’ adapter, a 8-nt barcode, a 10-nt pad sequence, a 2-nt linker and the 806 rB 16S rRNA gene-specific primer [74] (Table S3). PCR amplifications were performed in a final reaction volume of 25 µL containing 5 µL Green GoTaq^®^ reaction buffer (Promega, Madison, WI, USA), 0.5 µL 10 mM dNTPs (Promega), 0.5 µL 10 µM forward primer, 0.5 µL 10 µM reverse primer, 0.15 µL GoTaq^®^ DNA polymerase (5 U/µL, Promega) and 1 µL template DNA (0.1–10 ng/µL). The PCR program consisted of: initial denaturation of 2 min at 95 °C; 30 cycles of denaturation at 95 °C for 20 s, annealing at 55 °C for 15 s, and extension at 72 °C for 2 min; and final extension at 72 °C for 10 min. Samples were amplified in triplicate, after which the reaction volumes were pooled and 5 µL combined solution was run on a 1% agarose gel to assess amplification success. Next, the PCR products were purified using the QIAquick PCR Purification Kit (Qiagen, Venlo, The Netherlands). DNA was eluted from the spin column with 10 µL distilled DNase/RNase-free water (Invitrogen, Waltham, MA, USA). The DNA concentration of the elute was measured with the Nanodrop 2000c (Thermo Fisher Scientific, Waltham, MA, USA). An equimolar mixture of PCR products from unique samples, i.e., a sample library, was prepared and run on a 1% agarose gel. The gel band at ~292 bp was extracted and purified using the ZymocleanTM Gel DNA Recovery Kit (Zymo Research, Irvine, CA, USA). Elution was done with distilled DNase/RNase-free water. The sample mixture was reduced in volume by vacuum drying, and subsequently sent for Illumina paired end MiSEQ sequencing (2 × 250 bp) at GATC Biotech (Konstanz, Germany). A negative control sample was also included in the sample library because weak amplification was regularly detected in PCRs without template. The 16S rRNA gene amplicon sequences were deposited in the ENA SRA database under accession number PRJEB31855 (Table S3).

### 4.4. Processing of 16S MiSEQ Data

The 16S rRNA gene reads were processed with the MiSEQ standard operating procedure [73] (https://www.mothur.org/wiki/MiSeq_SOP, accessed November 30, 2017). In brief, reads were quality-filtered, assembled into contigs, filtered for chimaeras with VSEARCH [75], and clustered into operational taxonomic units (OTUs) based on a 97% identity threshold. The OTUs were annotated with the Ribosomal Database Project Classifier [76] using the SILVA SSU NR 128 database as a reference [77]. OTUs that were detected at a higher relative abundance in the negative control sample than in our biological samples were removed from the OTU table because they were assumed to be contamination.

### 4.5. Cultivation of Bacteria and Their Biomass Extraction

All 127 strains were preliminary screened with the agar plug diffusion assay against environmental bacteria (*Acinetobacter soli*, *Acinetobacter pitii*, *Aliagarivorans marinus*, *Aurantimonas coralicida*, *Exiguobacterium profundum*, *Microbulbifer variabilis*, *Pantoea eucrina*, *Pseudovibrio denitrificans*, *Rhodococcus corynebacterioides*, *Ruegeria areniliticus*, *Streptomyces flavoviridis*, and *Vibrio coralliilyticus*). Based on these results the 69 active strains were further cultured and extracted to be tested against human pathogenic microorganisms. Seed cultures were prepared by picking a bacterial colony from a 24 to 48 h old marine agar plate into a 15 mL Falcon tube holding 10 mL of liquid Marine Broth media (Carl Roth, Karlsruhe, Germany). After 3 days, 1 mL of this starting culture was transferred to 250 mL Erlenmeyer flask holding 100 mL of fresh Marine Broth media. All flasks were incubated at room temperature (about 23 °C) for 10 days to assure that the cultures reached the late stationary phase. Broth cultures were extracted with ethyl acetate (EtOAc; HPLC grade VWR International GmbH, Hannover, Germany) using Ultra-Turrax T65 (IKA, Staufen, Germany) at 12.000 rpm for 30 s with a broth culture: EtOAc partition of 1:2 (v/v). EtOAc extracts were separated from the aqueous phase by using a separation funnel. Afterwards the organic phase was evaporated in the rotary evaporator and stored in the −20 °C freezer until further analysis. 

### 4.6. Antimicrobial Assay

The panel of test microorganisms consisted of the following bacteria: Gram-negative bacteria *Escherichia coli* (DSM 1116) and *Pseudomonas aeruginosa* (PA16), Gram-positive bacteria *Bacillus subtilis* (DSM 10), *Staphylococcus aureus* (DSM 346), *Mycobacterium smegmatis* (ATCC 7000048), yeast *Candida albicans* (DSM 1665), *Rhodotorula glutinis* (DSM 10134) and filamentous fungi *Mucor hiemalis* (DSM 2656). A total of 20 µL raw extract 1 mg/mL and 180 µL bacterial/fungal suspension was tested in seven 1:2 serial dilution steps (dilution steps A to H) in 96-well plates for tissue cultures (TPP). Bacteria were cultivated in Mueller-Hinton bouillon (Roth) and fungi/yeasts in MYC medium (1.0% phytone peptone, 1.0% glucose, and 1.19% HEPES, pH 7.0). Start OD_600_ was 0.01 for *B. subtilis*, *E. coli* and *S. aureus*; start OD_548_ was 0.1 for *M. hiemalis*, *C. albicans*, *R. glutinis*, *M. smegmatis* and *P. aeruginosa*. The test organisms were cultivated at 30 °C and 160 rpm overnight. In this study, we use letter A-H for showing the different serial dilution steps of 1:2 (A: starting concentration-first well; B: first 1:2 dilution step–second well,...H: final dilution–last well). So, the highest bioactivity of the extract is the highest dilution (highest letter) in which the well still showed activity. Extracts showing activity in at least three wells were fractionated with semi-preparative high-performance liquid chromatography (HPLC) for peak-activity correlation. HPLC conditions were as described in [78]. Every 30 s 150 µL extract were collected in a new well of 96-well plates. After fractionation, the dried plate (N_2_) was inoculated with the former inhibited test organism (150 µL/well) and incubated overnight. The inhibited wells could be correlated with peaks/retention time/UV-spectrum in the chromatogram. Active extracts were further analyzed by UPLC-HRMS.

### 4.7. Inhibitory Effects on Hepatitis C Virus (HCV) Infectivity

Huh7.5 cells stably expressing Firefly luciferase (Huh7.5 Fluc) were cultured in Dulbecco’s modified minimum essential medium (DMEM, Gibco, Thermo Fisher Scientific, Schwerte, Germany) containing 2 mM L glutamine, 1% minimum essential medium nonessential amino acids (MEM NEAA, Gibco, Thermo Fisher Scientific, Schwerte, Germany), 100 μg/mL streptomycin, 100 IU/mL penicillin (Gibco, Thermo Fisher Scientific, Schwerte, Germany), 5 μg/mL blasticidin and 10% fetal bovine serum. Cells were maintained in a 37 °C environment with 5% CO_2_ supply. Cells were infected with Jc1-derived *Renilla* reporter viruses in the presence or absence of compounds as described [44]. Infected cells were lysed and then frozen at −80 °C for 1 h following measurements of *Renilla* and *Firefly luciferase* activities on a Berthold Technologies Centro XS3 Microplate Luminometer (Bad Wildbad, Germany) as indicators of viral genome replication and cell viability, respectively.

### 4.8. Chemical Analysis of Bioactive Extracts

The identification of bioactive and potential novel secondary metabolites was detected by UPLC-HRMS to obtain MS^1^ and MS^2^ data from the fraction of bioactive extract. MS^1^ were obtained from analysis of the HPLC fraction of bioactive extracts on UPLC-HRMS (MaXis ESI TOF, Bruker Daltonik GmbH, Bremen, Germany) using BEH C_18_ column (Waters ACQUITY, Milford, MA, USA) (1.7 µm 2.1 × 50 mm). A linear gradient from 95% H_2_O and 5% MeCN to 5%H_2_O and 95% MeCN was used. The buffer system was acetic acid and 5 mM ammonium acetate. Eluent was detected by ESI-MS monitoring *m*/*z* 50–2000. Peaks were analyzed using software Bruker Data Analysis 4.2 (Bruker Daltonik GmbH, Bremen, Germany). The exact mass of the detected compounds was then compared to various databases namely Marinlit (Royal Society of Chemistry, Cambridge, UK), Dictionary of Natural Product (ChemNetBase, Taylor&Francis, Abingdon, UK), METLIN (Scripps Research, La Jolla, CA, USA), and GNPS (University of California San Diego (UCSD), La Jolla, CA, USA) with a search variance of ± 0.01–0.005 Dalton. This method enables a rapid detection of putatively novel bioactive compounds prior to time-consuming compound isolation. 

MS^2^ were obtained in UPLC-HRMS (Waters Synapt G2-Si, Milford, MA, USA) with the same system as mentioned above. Peaks were analyzed using the software Waters MassLynx V4.1 (Milford, MA, USA). In this study, masses were measured in the positive mode. We analyzed the MS^2^ data by using METLIN and MASST GNPS to identify the likely fragments of the compounds or its analogues. Search parameter in METLIN MS/MS fragment were precursor tolerance of 20 ppm, collision energy 40 eV, MS/MS tolerance 0.01 Da. The peaks were picked from the 30 highest peaks in the MS^2^ spectra before the precursor *m*/*z*. The MASST GNPS are open MS^2^ search engine like gene analysis in BLAST in NCBI [79]. Parameter for the analysis were minimum cosine score 0.3; minimum matched peaks 6; parent mass tolerance 2; fragment mass tolerance 0.2; search also analog; and search database was GNPS.

## 5. Conclusions

In this study we identified and analyzed the culturable bacteria from just two different sea cucumbers. Analysis of the mass spectrometry data from the highly active fractions resulted to the identification of 23 precursor ions that either putatively known or partly identified and two unidentified precursors. Thus, our data corroborates that the screening approach for new antibiotics from untapped marine sources is still a promising approach in the search for new anti-infectants. The finding of putatively novel bacteria species also provides further opportunities for the isolation of novel compounds during follow-up studies.

## Figures and Tables

**Table 1:** Summary of all isolated and identified bacteria grouped on a phylum level as well as its source of isolation.

Phylum	*Holothuria leucospilota* (HL)		*Stichopus vastus* (SV)		TOTAL
	Internal Part	External Part	Internal Part	External Part	
Actinobacteria	23	19	3	18	63
Firmicutes	5	8	6	14	33
Proteobacteria	11	10	1	8	30
Bacteroidetes	-	-	-	1	1
TOTAL	39	37	10	41	127

**Table 2:** Bacteria isolated from *Holothuria leucocpilota* (HL) and *Stichopus vastus* (SV). Closest type strain based on the NCBI database, accession and strain number, % similarity to the closest type strain, sequence length of the 16S rRNA-gene sequence, sample origin and antimicrobial activity are provided. Here, bacteria were considered as putatively new bacteria species if they had a sequence similarity of less than 98% and considered as new genus if the sequence similarity was less than 95%. The letter in parentheses in the antimicrobial column indicates the level of activity based on the last active location in the test-well (A–H) in 1:2 serial dilutions.

No.	Next Related Type Strain	Sample Accession Number	Type Strain Accession Number	Similarity to Type Strain (%)	Sequence Length (bp)	Sample Name	Antimicrobial Activity	
							Antibacteria	Antifungal
**Phylum Actinobacteria**								
1.	*Brevibacterium luteolum*	MK696423	NR_114872.1	99.63	1076	SV 4 (ext)	-	-
2.	*Cellulosimicrobium funkei*	MK696437	NR_042937.1	99.78	915	HL 61 (ext)	-	-
3.	*Corynebacterium pilbarense*	MK696498	NR_116953.1	98.74	829	HL 119 (ext)	-	-
4.	*Dermacoccus nishinomiyaensis*	MK696488	NR_044872.1	99.72	1063	HL 57 (ext)	Bs (B), Sa (C)	Rg (A), Mh (A)
5.	*Dermacoccus profundi*	MK696484	NR_043262.1	99.89	1076	HL 11 (int)	-	-
6.	*Dermacoccus profundi*	MK696494	NR_043262.1	99.72	916	SV 127 (ext)	-	-
7.	*Dietzia maris*	MK696467	NR_118596.1	98.84	1126	SV 164b (ext)	Sa (A)	-
8.	*Glutamicibacter* sp.* (*G. nicotianae*)	MK696438	NR_026190.1	96.38	1056	HL 108 (ext)	Bs (B), Sa (A)	Rg (A)
9.	*Isoptericola chiayiensis*	MK696432	NR_116696.1	98.88	894	HL 44 (ext)	Bs (A)	-
10.	*Janibacter alkaliphilus*	MK696433	NR_109453.1	98.92	1018	SV 51 (ext)	Bs (A)	-
11.	*Janibacter anophelis*	MK696442	NR_043218.1	99.15	1062	HL 24 (int)	-	-
12.	*Janibacter melonis*	MK696486	NR_025805.1	99.79	964	HL 40 (int)	Bs (A)	-
13.	*Kocuria flava*	MK696544	NR_044308.1	99.21	892	HL 55 (int)	Bs(E), Ec (A), Sa (D)	-
14.	*Kocuria palustris*	MK696435	NR_026451.1	99.90	1045	HL 6 (ext)	Bs (C), Sa (C)	Rg (A)
15.	*Kocuria palustris*	MK696424	NR_026451.1	100.00	956	HL 7 (ext)	Bs (B)	-
16.	*Kocuria palustris*	MK696425	NR_026451.1	100.00	922	HL 8 (ext)	Bs (A)	-
17.	*Kocuria palustris*	MK696524	NR_026451.1	99.81	879	HL 60 (ext)	-	-
18.	*Kocuria palustris*	MK696426	NR_026451.1	100.00	1021	HL 12 (int)	Bs (B)	-
19.	*Kocuria palustris*	MK696522	NR_026451.1	99.71	917	HL 42 (int)	-	-
20.	*Kocuria palustris*	MK696441	NR_026451.1	98.76	913	SV 14 (ext)	-	-
21.	*Kytococcus sedentarius*	MK696431	NR_074714.2	99.88	1041	HL 30 (int)	Bs (A)	-
22.	*Kytococcus sedentarius*	MK696446	NR_074714.2	99.72	838	HL 43 (int)	-	-
23.	*Kytococcus sedentarius*	MK696483	NR_074714.2	99.72	980	SV 2 (ext)	Bs (B)	-
24.	*Micrococcus aloeverae*	MK696444	NR_134088.1	99.78	1041	HL 33 (ext)	-	-
25.	*Micrococcus aloeverae*	MK696430	NR_134088.1	99.79	937	HL 29 (int)	Bs (A)	-
26.	*Micrococcus aloeverae*	MK696436	NR_134088.1	99.52	918	SV 5 (ext)	Bs (A)	Rg (A)
27.	*Micrococcus aloeverae*	MK696523	NR_134088.1	99.36	908	SV 52 (ext)	-	-
28.	*Micrococcus endophyticus*	MK696473	NR_044365.1	98.62	1018	HL 261 (int)	-	-
29.	*Micrococcus flavus*	MK696517	NR_043881.1	99.20	1005	HL 237 (int)	Bs (B), Sa (B), Rg (B)	-
30.	*Micrococcus terreus*	MK696528	NR_116649.1	99.44	1081	SV 137 (ext)	-	-
31.	*Nocardioides* sp.* (*N. exalbidus*)	MK696451	NR_041526.1	97.96	1036	HL 111 (ext)	Bs (A), Sa (H)	-
32.	*Ornithinimicrobium kibberense*	MK696459	NR_043056.1	99.59	988	SV 135 (ext)	-	-
33.	New Genus of family Propionibacteriaceae * (*Pseudopropionibacterium profundi*)	MK696480	NR_159102.1	93.29	1047	SV 17 (ext)	-	-
34.	*Rothia kristinae*	MK696477	NR_026199.1	99.29	989	HL 37 (ext)	-	-
35.	*Serinicoccus* sp.* (*S. profundi*)	MK696482	NR_116387.1	97.91	719	SV 16 (ext)	-	-
36.	*Streptomyces cavourensis*	MK696479	NR_043851.1	100.00	1034	SV 21 (int)	Bs (H), Sa (E)	Mh (G)
**Phylum Firmicutes**								
1.	*Bacillus aryabhattai*	MK696496	NR_115953.1	99.91	1134	HL 270 (int)	-	-
2.	*Bacillus cereus*	MK696514	NR_157734.1	99.91	1132	HL 229 (int)	-	-
3.	*Bacillus idriensis*	MK696468	NR_043268.1	99.47	948	HL 251 (int)	-	-
4.	*Bacillus safensis*	MK696463	NR_041794.1	100.00	927	SV 147 (ext)	Bs (B), Sa (H)	-
5.	*Bacillus safensis*	MK696525	NR_113945.1	99.91	1126	HL 63 (ext)	Sa (H)	-
6.	New genus of family Bacillaceae (*Bacillus sonorensis*) *	MK696542	NR_113993.1	92.88	1081	HL 79 (ext)	Sa (A)	-
7.	*Staphylococcus arlettae*	MK696500	NR_024664.1	99.65	1149	SV 133 (ext)	-	-
8.	*Staphylococcus cohnii*	MK696452	NR_036902.1	99.80	990	HL 113 (ext)	-	-
9.	*Staphylococcus cohnii* subsp. *urealyticus*	MK696447	NR_037046.1	99.31	1121	HL 67 (ext)	Sa (H)	-
10.	*Staphylococcus cohnii* subsp. *urealyticus*	MK696422	NR_037046.1	100.00	1116	SV 1 (ext)	Bs (B)	-
11.	*Staphylococcus cohnii* subsp. *urealyticus*	MK696458	NR_037046.1	99.73	953	SV 131 (ext)	-	-
12.	*Staphylococcus cohnii* subsp. *urealyticus*	MK696440	NR_037046.1	99.80	1098	SV 144 (ext)	Sa (A)	-
13.	*Staphylococcus edaphicus*	MK696526	NR_156818.1	99.73	1108	HL 75 (ext)	Bs (C), Sa (B)	Rg (B)
14.	*Staphylococcus haemolyticus*	MK696532	NR_113345.1	99.33	1044	SV 183 (ext)	-	-
15.	*Staphylococcus pasteuri*	MK696531	NR_114435.1	99.91	1117	SV 173 (ext)	-	-
16.	*Staphylococcus warneri*	MK696543	NR_025922.1	99.91	1084	HL 100 (ext)	Sa (A)	-
**Phylum Proteobacteria**								
1.	*Acinetobacter schindleri*	MK696475	NR_025412.1	98.95	1047	HL 265 (int)	-	-
2.	*Epibacterium mobile*	MK696445	NR_114024.1	99.64	1112	HL 38 (ext)	Bs (B)	-
3.	*Erythrobacter vulgaris*	MK696434	NR_043136.1	99.18	980	SV 54 (int)	Ec (A)	-
4.	*Erythrobacter vulgaris*	MK696478	NR_043136.1	99.36	937	HL 45 (ext)	Bs (A)	-
5.	*Pantoea septica*	MK696487	NR_116752.1	99.14	1080	SV 138 (ext)	Bs (A)	-
6.	New genus of family Rhodobacteraceae (*Paracoccus beibuensis*)	-	NR_116400.1	93.08	1011	SV 155 (ext)	Sa (E)	-
7.	*Paracoccus* sp.* (*P. koreensis*)	MK696429	NR_114060.1	97.33	940	HL 28 (int)	Bs (A)	Mh (A)
8.	*Paracoccus marinus*	MK696491	NR_113921.1	99.03	928	HL 256 (int)	Bs (A), Sa (A)	-
9.	*Paracoccus sulfuroxidans*	MK696428	NR_043887.1	98.25	861	HL 27 (int)	-	-
10.	*Pseudomonas stutzeri*	MK696497	NR_041715.1	99.21	1079	HL 26 (int)	Bs (B)	-
11.	*Psychrobacter celer*	MK696489	NR_043225.1	99.27	1100	HL 58 (ext)	-	-
12.	*Psychrobacter marincola*	MK696539	NR_025458.1	99.40	1165	HL 72 (ext)	Sa (A)	-
13.	*Vibrio alginolyticus*	MK696427	NR_118258.1	99.52	1039	HL 22 (ext)	Bs (G), Sa (E), Ms (A)	Rg (B), Mh (B)
14.	*Vibrio* sp.* (*V. harveyi*)	MK696456	NR_043165.1	96.23	1074	HL 125 (ext)	Bs (A), Sa (A)	-
15.	*Vibrio harveyi*	MK696454	NR_113784.1	99.80	996	HL 121 (ext)	Sa (A)	Rg (A)
16.	*Vibrio owensii*	MK696449	NR_117424.1	99.34	907	HL 107 (ext)	-	-
17.	*Vibrio owensii*	MK696499	NR_117424.1	99.44	1082	HL 122 (ext)	Sa (A)	Rg (A)

(*): represent new bacterial species (closest match in NCBI database). Bs: *Bacillus subtilis*, Ec: *Escherichia coli*, Mh: *Mucor hiemalis*, Rg: *Rhodotorula glutinis*, Sa: *Staphylococcus aureus*; HL: *Holothuria leucocpilota*; SV: *Stichopus vastus*; -: not active; NT: Not tested; (int): isolated from internal part, (ext): isolated from external part.

**Table 3:** The summary of 25 precursor/parent (MS^1^) as well as product/daughter (MS^2^) ion analysis from the bioactive bacterial strains. Exact masses from HRMS analysis (±0.005 Da) were compared with known databases (MarinLit, DNP, METLIN and GNPS). The MS^2^ data were compared with available library from the public databases (METLIN and Mass Spectrometry Search Tool (MASST) in GNPS).

Strain	Precursor Ions (*m*/*z*)	Finding Match Compounds Based on MS^1^ in Databases (M ± 0.005)	Finding Match Compounds Based on MS^2^ in Databases (incl. Analog) *	Annotation
*Streptomyces cavourensis* SV 21	458.181 [M + H]^+^ (*M* = *457.173*)	F	LV	Partly identified with low match value
	490.207 [M + H]^+^ (*M* = *489.200*)	F	LV	Partly identified with low match value
	1128.665 [M + NH_4_]^+^ (*M* = *1110.630*)	F	F	Putative Valinomycin *
	1142.678 [M + NH_4_]^+^ (*M* = *1124.644*)	NF	F	Partly identified as valinomycin derivate *
	663.454 [M + H]^+^ (*M* = *662.447*)	F	LV	Partly identified with low match value
*Kocuria flava* HL 55	1140.219 [M + H]^+^ (*M* = *1139.211*)	NF	NF	unidentified
	1515.373 [M + H]^+^ (*M* = *1514.366*)	F	F	Putative kocurin *
*Bacillus safensis* HL 63 and *Staphylococcus cohnii* subsp. *urealyticus* HL 67	1070.643 [M + H]^+^ (*M* = *1069.636*)	NF	F	Putative surfactins
	1102.616 [M + H]^+^ (*M* = *1101.609*)	F	F	Putative surfactins
	1076.629 [M+Na]^+^ (*M* = *1053.640*)	NF	F	Putative surfactins
	1068.661 [M + H]^+^ (*M* = *1067.654*)	F	F	Putative surfactins
	1022.674 [M + H]^+^ (*M* = *1021.667*)	F	F	Putative surfactins
	1058.671 [M + Na]^+^ (*M* = *1035.684*)	F	F	Putative surfactins *
	1072.686 [M + Na]^+^ (*M* = *1049.698*)	F	F	Putative surfactins *
	1096.692 [M + H]^+^ (*M* = *1095.685*)	F	F	Putative surfactins
	1086.702 [M + Na]^+^ (*M* = *1063.714*)	F	F	Putative surfactins
	875.534 [M + Na]^+^ (*M* = *852.545*)	NF	NF	unidentified
	1100.717 [M + Na]^+^ (*M* = *1077.723*)	F	F	Putative surfactins
*Staphylococcus edaphicus* HL 75	347.212 [M + H]^+^ (*M* = *346.205*)	F	LV	Partly identified with low match value
	395.213 [M + H]^+^ (*M* = *394.206*)	F	LV	Partly identified with low match value
*Bacillus safensis* SV 147, SV 155 (putatively new genus of Rhodobacteraceae), and *Nocardioides* sp. HL 111	1336.478 [M + H]^+^ (*M* = *1335.471*)	F	F	Putative plantazolicin A *
	1044.657 [M + Na]^+^ (*M* = *1021.668*)	F	F	Putative surfactins
	1058.671 [M + Na]^+^ (*M* = *1035.683*)	F	F	Putative surfactins
	1050.705 [M + H]^+^ (*M* = *1049.698*)	F	F	Putative surfactins
	1086.703 [M + Na]^+^ (*M* = *1063.713*)	F	F	Putative surfactins

F = Found; LV = low match value; NF = Not Found. * MS^2^ spectra are given as examples for compounds marked with a star (cf. Figures S5–S12).

## Supplementary Materials

The following are available online at https://www.mdpi.com/1660-3397/17/11/635/s1. Figure S1: 16S rRNA gene-based phylogeny of Actinobacteria diversity, Figure S2: 16S rRNA gene-based phylogeny of Firmicutes diversity, Figure S3: 16S rRNA gene-based phylogeny of Proteobacteria, Figure S4. Viability of the liver cells in anti-HCV assay, Figure S5: Identification of precursor from *Streptomyces cavourensis* SV 21 with *m*/*z* 1128.637 [M + NH4]+, Figure S6: Identification of precursor from *Streptomyces cavourensis* SV 21 with *m*/*z* 1142.678 [M + NH4]+, Figure S7: MS1 and MS2 spectra of precursor in *Kocuria flava* HL 55 with *m*/*z* 1140.219 [M + H]+, Figure S8: MS1 and MS2 spectra of precursor in *Kocuria flava* HL 55 with *m*/*z* 1515.373 [M + H]+, Figure S9: Identification of precursor from *Bacillus safensis* HL 63 and *Staphylococcus cohnii* subsp. *urealyticus* HL 67 with *m*/*z* 1058.671 [M + Na]+, Figure S10: Identification of precursor from *Bacillus safensis* HL 63 and *Staphylococcus cohnii* subsp. *urealyticus* HL 67 with *m*/*z* 1072.686 [M + Na]+, Figure S11: MS1 and MS2 spectra of precursor in *Bacillus safensis HL 63* with *m*/*z* 875.538 [M + Na]+, Figure S12: MS1 and MS2 spectra of precursor in *Bacillus safensis* SV. 147, *Paracoccus beibuensis* SV. 155, and *Nocardioides exalbidus* HL. 111 with *m*/*z* 1336.478 [M + H]+, Table S1: Total bacteria isolated from *Holothuria leucocpilota* (HL) and *Stichopus vastus* (SV), Table S2: Search results of the precursor molecular ions and its exact masses, Table S3: This table lists the samples that were analyzed by 16S amplicon sequencing, the primers were used per sample, and the accession numbers under which the amplicon datasets can be found in the ENA SRA database (project number: PRJEB31855), Table S4: Full-length sequence of 16S rRNA from SV 155.

## References

[1] Bordbar S., Anwar F., Saari N. (2011). High-value components and bioactives from sea cucumbers for functional foods—A review. Mar. Drugs.

[2] Guo Y., Ding Y., Xu F., Liu B., Kou Z., Xiao W., Zhu J. (2015). Systems pharmacology-based drug discovery for marine resources: An example using sea cucumber (Holothurians). J. Ethnopharmacol..

[3] Masre S.F., Yip G.W., Sirajudeen K.N.S., Ghazali F.C. (2010). Wound healing activity of total sulfated glycosaminoglycan (GAG) from *Stichopus vastus* and *Stichopus hermanni* integumental tissue in rats. Int. J. Mol. Med. Adv. Sci..

[4] Abedin M.Z., Karim A.A., Latiff A.A., Gan C.-Y., Ghazali F.C., Zzaman W., Hossain M.M., Ahmed F., Absar N., Sarker M.Z.I. (2014). Physicochemical and biochemical properties of pepsin-solubilized collagen isolated from the integument of sea cucumber (*Stichopus vastus*). J. Food Process. Preserv..

[5] Abedin M., Karim A., Gan C., Ghazali F., Barzideh Z., Zzaman W., Zaidul I. (2015). Identification of angiotensin I converting enzyme inhibitory and radical scavenging bioactive peptides from sea cucumber (*Stichopus vastus*) collagen hydrolysates through optimization. Int. Food Res. J..

[6] Conand C., Purcell S., Gamboa R. (2013). Holothuria leucospilota. The IUCN Red List of Threatened Species.

[7] Baharara J., Amini E., Vazifedan V. (2016). Concomitant use of sea cucumber organic extract and radiotherapy on proliferation and apoptosis of cervical (HeLa) cell line. Zahedan J. Res. Med. Sci..

[8] Baharara J., Amini E., Nikdel N., Salek-Abdollahi F. (2016). The cytotoxicity of dacarbazine potentiated by sea cucumber saponin in resistant B16F10 melanoma cells through apoptosis induction. Avicenna J. Med. Biotechnol..

[9] Soltani M., Parivar K., Baharara J., Kerachian M.A., Asili J. (2014). Hemolytic and cytotoxic properties of saponin purified from *Holothuria leucospilota* sea cucumber. Rep. Biochem. Mol. Biol..

[10] Soltani M., Parivar K., Baharara J., Kerachian M.A., Asili J. (2015). Putative mechanism for apoptosis-inducing properties of crude saponin isolated from sea cucumber (*Holothuria leucospilota*) as an antioxidant compound. Iran. J. Basic Med. Sci..

[11] Pangestuti R., Arifin Z. (2018). Medicinal and health benefit effects of functional sea cucumbers. J. Tradit. Complement. Med..

[12] Farjami B., Nematollahi M.A., Moradi Y., Irajian G., Nazemi M., Ardebili A., Pournajaf A. (2013). Antibacterial activity of the sea cucumber *Holothuria leucospilota*. Int. J. Mol. Clin. Microbiol..

[13] Adibpour N., Nasr F., Nematpour F., Shakouri A., Ameri A. (2014). Antibacterial and antifungal activity of *Holothuria leucospilota* isolated from Persian Gulf and Oman Sea. Jundishapur J. Microbiol..

[14] Proksch P., Edrada R., Ebel R. (2002). Drugs from the seas–current status and microbiological implications. Appl. Microbiol. Biotechnol..

[15] Haygood M.G., Schmidt E.W., Davidson S.K., Faulkner D.J. (1999). Microbial symbionts of marine invertebrates: Opportunities for microbial biotechnology. J. Mol. Microbiol. Biotechnol..

[16] Osinga R., Armstrong E., Burgess J.G., Hoffmann F., Reitner J., Schumann-Kindel G. (2001). Sponge–microbe associations and their importance for sponge bioprocess engineering. Hydrobiologia.

[17] Müller R., Wink J. (2014). Future potential for anti-infectives from bacteria—How to exploit biodiversity and genomic potential. Int. J. Med. Microbiol..

[18] Stadler M., Dersch P. (2017). How to Overcome the Antibiotic Crisis.

[19] Pietschmann T., Brown R.J. (2019). Hepatitis C virus. Trends Microbiol..

[20] Roy V., King L. (2016). Betting on hepatitis C: How financial speculation in drug development influences access to medicines. Bmj.

[21] Thomas T.R.A., Kavlekar D.P., LokaBharathi P.A. (2010). Marine drugs from sponge-microbe association—A review. Mar. Drugs.

[22] Moitinho-Silva L., Nielsen S., Amir A., Gonzalez A., Ackermann G.L., Cerrano C., Astudillo-Garcia C., Easson C., Sipkema D., Liu F. (2017). The sponge microbiome project. Gigascience.

[23] Steinert G., Rohde S., Janussen D., Blaurock C., Schupp P.J. (2017). Host-specific assembly of sponge-associated prokaryotes at high taxonomic ranks. Sci. Rep..

[24] Steinert G., Taylor M.W., Deines P., Simister R.L., de Voogd N.J., Hoggard M., Schupp P.J. (2016). In four shallow and mesophotic tropical reef sponges from Guam the microbial community largely depends on host identity. PeerJ.

[25] Steinert G., Taylor M.W., Schupp P.J. (2015). Diversity of actinobacteria associated with the marine ascidian Eudistoma toealensis. Mar. Biotechnol..

[26] Tawfike A., Attia E.Z., Desoukey S.Y., Hajjar D., Makki A.A., Schupp P.J., Edrada-Ebel R., Abdelmohsen U.R. (2019). New bioactive metabolites from the elicited marine sponge-derived bacterium *Actinokineospora spheciospongiae* sp. nov. AMB Express.

[27] Thomas T., Moitinho-Silva L., Lurgi M., Björk J.R., Easson C., Astudillo-García C., Olson J.B., Erwin P.M., López-Legentil S., Luter H. (2016). Diversity, structure and convergent evolution of the global sponge microbiome. Nat. Commun..

[28] Steinert G., Whitfield S., Taylor M.W., Thoms C., Schupp P.J. (2014). Application of diffusion growth chambers for the cultivation of marine sponge-associated bacteria. Mar. Biotechnol..

[29] Gao F., Li F., Tan J., Yan J., Sun H. (2014). Bacterial community composition in the gut content and ambient sediment of sea cucumber *Apostichopus japonicus* revealed by 16S rRNA gene pyrosequencing. PLoS ONE.

[30] Böhringer N., Fisch K.M., Schillo D., Bara R., Hertzer C., Grein F., Eisenbarth J.-H., Kaligis F., Schneider T., Wägele H. (2017). Antimicrobial potential of bacteria associated with marine sea slugs from North Sulawesi, Indonesia. Front. Microbiol..

[31] Tangerina M.M., Correa H., Haltli B., Vilegas W., Kerr R.G. (2017). Bioprospecting from cultivable bacterial communities of marine sediment and invertebrates from the underexplored Ubatuba region of Brazil. Arch. Microbiol..

[32] Zhang X., Nakahara T., Miyazaki M., Nogi Y., Taniyama S., Arakawa O., Inoue T., Kudo T. (2012). Diversity and function of aerobic culturable bacteria in the intestine of the sea cucumber *Holothuria leucospilota*. J. Gen. Appl. Microbiol..

[33] Dupont S., Carre-Mlouka A., Domart-Coulon I., Vacelet J., Bourguet-Kondracki M.-L. (2014). Exploring cultivable bacteria from the prokaryotic community associated with the carnivorous sponge *Asbestopluma hypogea*. FEMS Microbiol. Ecol..

[34] Austin B., Zhang X.H. (2006). Vibrio harveyi: A significant pathogen of marine vertebrates and invertebrates. Lett. Appl. Microbiol..

[35] Zhang J., Cao Z., Li Z., Wang L., Li H., Wu F., Jin L., Li X., Li S., Xu Y. (2015). Effect of bacteriophages *onVibrio alginolyticusInfection* in the sea cucumber, *Apostichopus japonicus* (Selenka). J. World Aquac. Soc..

[36] Huang J., Zeng B., Liu D., Wu R., Zhang J., Liao B., He H., Bian F. (2018). Classification and structural insight into vibriolysin-like proteases of *Vibrio* pathogenicity. Microb. Pathog..

[37] Wang X., Huang Y., Sheng Y., Su P., Qiu Y., Ke C., Feng D. (2017). Antifouling activity towards mussel by small-molecule compounds from a strain of *Vibrio alginolyticus* bacterium associated with sea anemone *Haliplanella* sp.. J. Microbiol. Biotechnol..

[38] Lateef A., Adelere I.A., Gueguim-Kana E.B. (2015). The biology and potential biotechnological applications of *Bacillus safensis*. Biologia.

[39] Gesheva V., Vasileva-Tonkova E. (2012). Production of enzymes and antimicrobial compounds by halophilic Antarctic *Nocardioides* sp. grown on different carbon sources. World J. Microbiol. Biotechnol..

[40] Pan H.Q., Yu S.Y., Song C.F., Wang N., Hua H.M., Hu J.C., Wang S.J. (2015). Identification and characterization of the antifungal substances of a novel *Streptomyces cavourensis* NA4. J. Microbiol. Biotechnol..

[41] Begum I.F., Mohankumar R., Jeevan M., Ramani K. (2016). GC–MS analysis of bio-active molecules derived from *Paracoccus pantotrophus* FMR19 and the antimicrobial activity against bacterial pathogens and MDROs. Indian J. Microbiol..

[42] Zhang F., Ye Q., Chen Q., Yang K., Zhang D., Chen Z., Lu S., Shao X., Fan Y., Yao L. (2018). Algicidal activity of novel marine bacterium *Paracoccus* sp. strain Y42 against a harmful algal-bloom-causing dinoflagellate, *Prorocentrum donghaiense*. Appl. Environ. Microbiol..

[43] Lukman A.L., Nordin N.F.H., Kamarudin K.R. (2014). Microbial population in the coelomic fluid of *Stichopus chloronotus* and *Holothuria* (*Mertensiothuria*) *leucospilota* collected from Malaysian waters. Sains Malays..

[44] Ciesek S., von Hahn T., Colpitts C.C., Schang L.M., Friesland M., Steinmann J., Manns M.P., Ott M., Wedemeyer H., Meuleman P. (2011). The green tea polyphenol, epigallocatechin-3-gallate, inhibits hepatitis C virus entry. Hepatology.

[45] Matloub A.A., Gomaa E.Z., Hassan A.A., Elbatanony M.M., El-Senousy W.M. (2019). Comparative chemical and bioactivity studies of intra-and extracellular metabolites of endophytic bacteria, *Bacillus subtilis* NCIB 3610. Int. J. Pept. Res. Ther..

[46] Covington B.C., McLean J.A., Bachmann B.O. (2017). Comparative mass spectrometry-based metabolomics strategies for the investigation of microbial secondary metabolites. Nat. Prod. Rep..

[47] Yoshioka T., Watanabe A., Kojima I., Shimauchi Y., Okabe M., Fukagawa Y., Ishikura T. (1984). Structures of OA-6129D and E, new carbapenam antibiotics. J. Antibiot..

[48] Paulo B.S., Sigrist R., Angolini C.F., de Oliveira L.G. (2019). New cyclodepsipeptide derivatives revealed by genome mining and molecular networking. ChemistrySelect.

[49] Callow R., Taylor D. (1952). 429. The cardio-active glycosides of *Strophanthus sarmentosus* P. DC.“sarmentoside B” and its relation to an original sarmentobioside. J. Chem. Soc..

[50] Martin J., da S Sousa T., Crespo G., Palomo S., Gonzalez I., Tormo J.R., de la Cruz M., Anderson M., Hill R.T., Vicente F. (2013). Kocurin, the true structure of PM181104, an anti-methicillin-resistant *Staphylococcus aureus* (MRSA) thiazolyl peptide from the marine-derived bacterium *Kocuria palustris*. Mar. Drugs.

[51] Liu X.-Y., Yang S.-Z., Mu B.-Z. (2009). Production and characterization of a C_15_-surfactin-O-methyl ester by a lipopeptide producing strain *Bacillus subtilis* HSO121. Process. Biochem..

[52] Kalyon B., Helaly S.E., Scholz R., Nachtigall J., Vater J., Borriss R., Süssmuth R.D. (2011). Plantazolicin A and B: Structure elucidation of ribosomally synthesized thiazole/oxazole peptides from *Bacillus amyloliquefaciens* FZB42. Org. Lett..

[53] Wright G.D. (2017). Opportunities for natural products in 21st century antibiotic discovery. Nat. Prod. Rep..

[54] Baltz R.H. (2008). Renaissance in antibacterial discovery from actinomycetes. Curr. Opin. Pharmacol..

[55] Schinke C., Martins T., Queiroz S.C.N., Melo I.S., Reyes F.G.R. (2017). Antibacterial compounds from marine bacteria, 2010–2015. J. Nat. Prod..

[56] Khotimchenko Y. (2018). Pharmacological potential of sea cucumbers. Int. J. Mol. Sci..

[57] Purcell S., Conand C., Uthicke S., Byrne M. (2016). Ecological roles of exploited sea cucumbers. Oceanography and Marine Biology: An Annual Review V54.

[58] Pereira D.M., Andrade P.B., Pires R.A., Reis R.L. (2014). Chemical Ecology of Echinoderms: Impact of Environment and Diet in Metabolomic Profile.

[59] Seipke R.F., Kaltenpoth M., Hutchings M.I. (2012). Streptomyces as symbionts: An emerging and widespread theme?. FEMS Microbiol. Rev..

[60] Kamarudin K.R., Ngah N., Hamid T.H.T.A., Susanti D. (2013). Isolation of a PIgment-producing strain of *Staphylococcus kloosii* from the respiratory tree of *Holothuria* (*Mertensiothuria*) *leucospilota* (Brandt 1835) from Malaysian waters. Trop. Life Sci. Res..

[61] Brockmann H., Schmidt-Kastner G. (1955). Valinomycin I, XXVII. Mitteil. über antibiotica aus actinomyceten. Chem. Ber..

[62] Pimentel-Elardo S.M., Kozytska S., Bugni T.S., Ireland C.M., Moll H., Hentschel U. (2010). Anti-parasitic compounds from *Streptomyces* sp. strains isolated from *Mediterranean sponges*. Mar. Drugs.

[63] Wu C.-Y., Jan J.-T., Ma S.-H., Kuo C.-J., Juan H.-F., Cheng Y.-S.E., Hsu H.-H., Huang H.-C., Wu D., Brik A. (2004). Small molecules targeting severe acute respiratory syndrome human coronavirus. Proc. Natl. Acad. Sci. USA.

[64] Cheng Y.Q. (2006). Deciphering the biosynthetic codes for the potent anti-SARS-CoV cyclodepsipeptide valinomycin in *Streptomyces tsusimaensis* ATCC 15141. ChemBioChem.

[65] Sabdono A., Sawonua P.H., Kartika A.G.D., Amelia J.M., Radjasa O.K. (2015). Coral diseases in Panjang Island, Java Sea: Diversity of anti–pathogenic bacterial coral symbionts. Procedia Chem..

[66] Scheubert K., Hufsky F., Petras D., Wang M., Nothias L.-F., Duehrkop K., Bandeira N., Dorrestein P.C., Boecker S. (2017). Significance estimation for large scale metabolomics annotations by spectral matching. Nat. Commun..

[67] Sottorff I., Wiese J., Lipfert M., Preußke N., Sönnichsen F.D., Imhoff J.F. (2019). Different secondary metabolite profiles of phylogenetically almost identical Streptomyces griseus strains originating from geographically remote locations. Microorganisms.

[68] Gontang E.A., Fenical W., Jensen P.R. (2007). Phylogenetic diversity of gram-positive bacteria cultured from marine sediments. Appl. Environ. Microbiol..

[69] Franklin R.B., Garland J.L., Bolster C.H., Mills A.L. (2001). Impact of dilution on microbial community structure and functional potential: Comparison of numerical simulations and batch culture experiments. Appl. Environ. Microbiol..

[70] Jiang H., Dong H., Zhang G., Yu B., Chapman L.R., Fields M.W. (2006). Microbial diversity in water and sediment of Lake Chaka, an athalassohaline lake in northwestern China. Appl. Environ. Microbiol..

[71] Stackebrandt E., Goebel B.M. (1994). Taxonomic note: A place for DNA-DNA reassociation and 16S rRNA sequence analysis in the present species definition in bacteriology. Int. J. Syst. Evol. Microbiol..

[72] Tindall B.J., Rosselló-Móra R., Busse H.-J., Ludwig W., Kämpfer P. (2010). Notes on the characterization of prokaryote strains for taxonomic purposes. Int. J. Syst. Evol. Microbiol..

[73] Kozich J.J., Westcott S.L., Baxter N.T., Highlander S.K., Schloss P.D. (2013). Development of a dual-index sequencing strategy and curation pipeline for analyzing amplicon sequence data on the MiSeq Illumina sequencing platform. Appl. Environ. Microbiol..

[74] Apprill A., McNally S., Parsons R., Weber L. (2015). Minor revision to V4 region SSU rRNA 806R gene primer greatly increases detection of SAR11 bacterioplankton. Aquat. Microb. Ecol..

[75] Rognes T., Flouri T., Nichols B., Quince C., Mahé F. (2016). VSEARCH: A versatile open source tool for metagenomics. PeerJ.

[76] Wang Q., Garrity G.M., Tiedje J.M., Cole J.R. (2007). Naive Bayesian classifier for rapid assignment of rRNA sequences into the new bacterial taxonomy. Appl. Environ. Microbiol..

[77] Quast C., Pruesse E., Yilmaz P., Gerken J., Schweer T., Yarza P., Peplies J., Glöckner F.O. (2012). The SILVA ribosomal RNA gene database project: Improved data processing and web-based tools. Nucleic Acids Res..

[78] Okanya P.W., Mohr K.I., Gerth K., Jansen R., Müller R. (2011). Marinoquinolines A−F, pyrroloquinolines from *Ohtaekwangia kribbensis* (Bacteroidetes). J. Nat. Prod..

[79] Wang M., Jarmusch A.K., Vargas F., Aksenov A.A., Gauglitz J.M., Weldon K., Petras D., Silva R.D., Quinn R., Melnik A.V. (2019). MASST: A web-based basic mass spectrometry search tool for molecules to search public data. BioRxiv.

