# Supplementary material for: Biotechnological Potential of Bacteria Isolated from the Sea Cucumber Holothuria leucospilota and Stichopus vastus from Lampung, Indonesia
Source: Mar Drugs. 2019 Nov 8;17(11):635. doi: 10.3390/md17110635 (PMC6891442; doi:10.3390/md17110635)
Supplement: Supplementary file 1 [file marinedrugs-17-00635-s001.pdf]

SUPPLEMENTARY DATA

Figure S1. 16S rRNA gene-based phylogeny of Actinobacteria diversity.

Figure S2. 16S rRNA gene-based phylogeny of Firmicutes diversity.

Figure S3. 16S rRNA gene-based phylogeny of Proteobacteria.

Figure S4. Viability of the liver cells in anti-HCV assay.

Figure S5. Identification of precursor from *Streptomyces cavourensis* SV 21 with *m*/*z* 1128.637 $[M+NH_4]^+$.

Figure S6. Identification of precursor from *Streptomyces cavourensis* SV 21 with *m*/*z* 1142.678 $[M+NH_4]^+$.

Figure S7. MS^1 and MS^2 spectra of precursor in *Kocuria flava* HL 55 with *m*/*z* 1140.219 $[M+H]^+$.

Figure S8. MS^1 and MS^2 spectra of precursor in *Kocuria flava* HL 55 with *m*/*z* 1515.373 $[M+H]^+$.

Figure S9. Identification of precursor from *Bacillus safensis* HL 63 and *Staphylococcus cohnii* subsp. *urealyticus* HL 67 with *m*/*z* 1058.671 $[M+Na]^+$.

Figure S10. Identification of precursor from *Bacillus safensis* HL 63 and *Staphylococcus cohnii* subsp. *urealyticus* HL 67 with *m*/*z* 1072.686 $[M+Na]^+$.

Figure S11. MS^1 and MS^2 spectra of precursor in *Bacillus safensis HL 63* with *m*/*z* 875.538 $[M+Na]^+$.

Figure S12. MS^1 and MS^2 spectra of precursor in *Bacillus safensis* SV. 147, *Paracoccus beibuensis* SV. 155, and *Nocardioides exalbidus* HL. 111 with *m*/*z* 1336.478 $[M+H]^+$.

Table S1. Total bacteria isolated from *Holothuria leucocpilota* (HL) and *Stichopus vastus* (SV).

Table S2. Search results of the precursor molecular ions and its exact masses.

Table S3. This table lists the samples that were analyzed by 16S amplicon sequencing, the primers were used per sample, and the accession numbers under which the amplicon datasets can be found in the ENA SRA database (project number: PRJEB31855).

Table S4. Full-length sequence of 16S rRNA from SV 155.

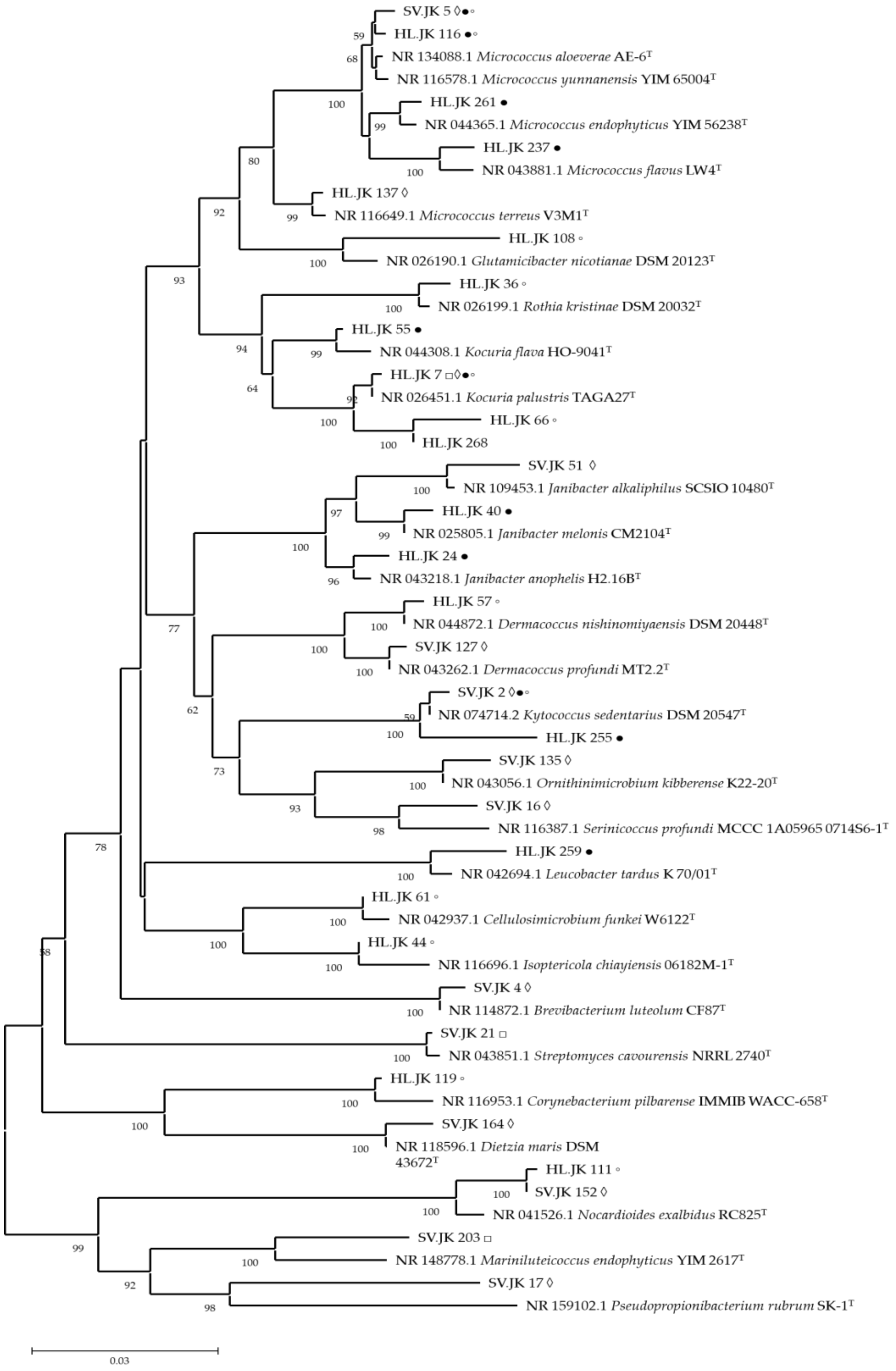

Figure S1. 16S rRNA gene-based phylogeny of Actinobacteria diversity associated with *Holothuria leucospilota* and *Stichopus vastus* (Neighbor-joining tree); ●: Bacterial source from internal part of *H. leucospilota* ; ◦: external part of *H. leucospilota* ; □: internal part of *S. vastus*; ◊: external part of *S. vastus*.

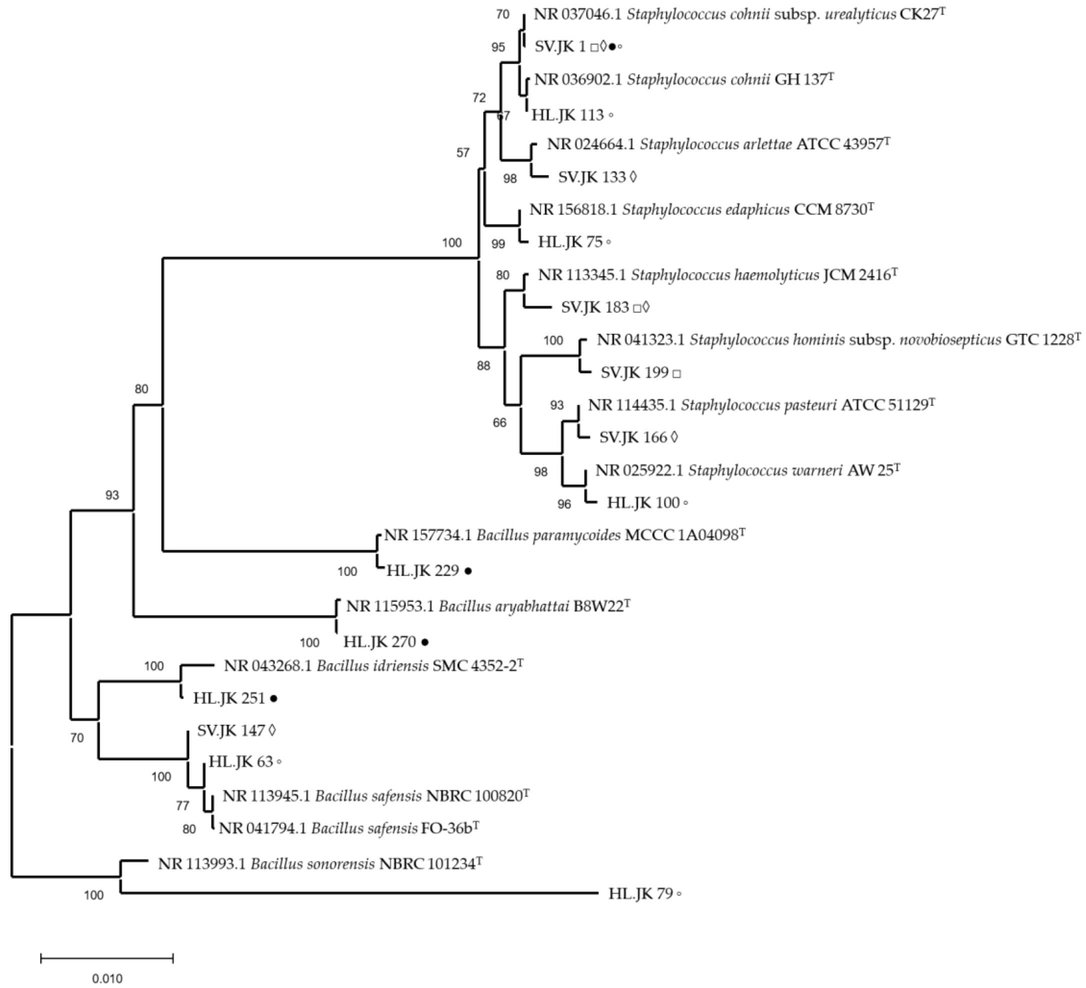

Figure S2. 16S rRNA gene-based phylogeny of Firmicutes diversity from *Holothuria leucospilota* and *Stichopus vastus* (Neighbor-joining tree); ●: Bacterial source from internal part of *H. leucospilota* ; ◦: external part of *H. leucospilota* ; □: internal part of *S. vastus;* ◊: external part of *S. vastus*.

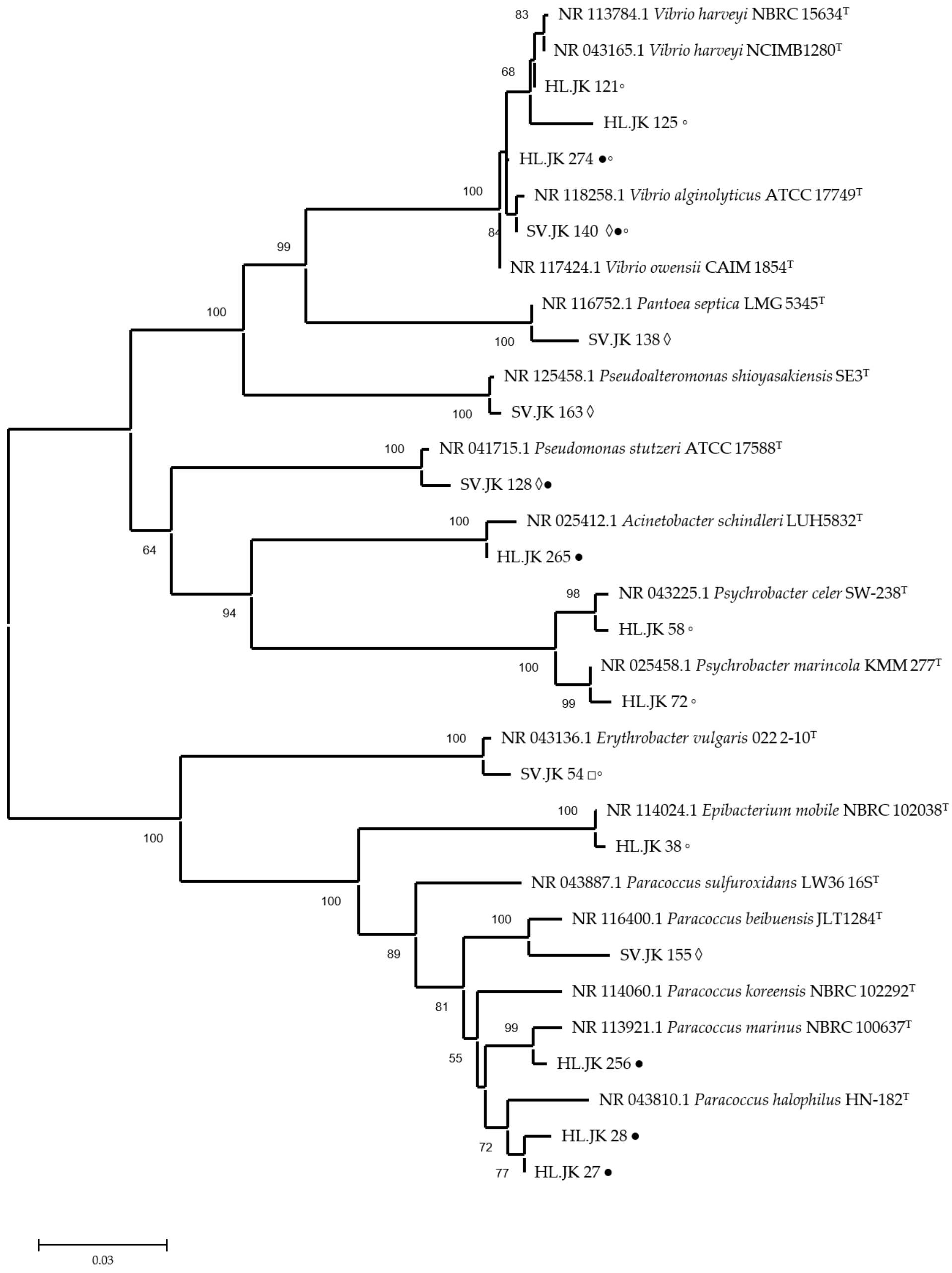

Figure S3. 16S rRNA gene-based phylogeny of Proteobacteria from *Holothuria leucospilota* and *Stichopus vastus* (Neighbor-joining tree)*;* ●: Bacterial source from internal part of *H. leucospilota* ; ◦: external part of *H. leucospilota* ; □: internal part of *S. vastus;* ◊: external part of *S. vastus*.

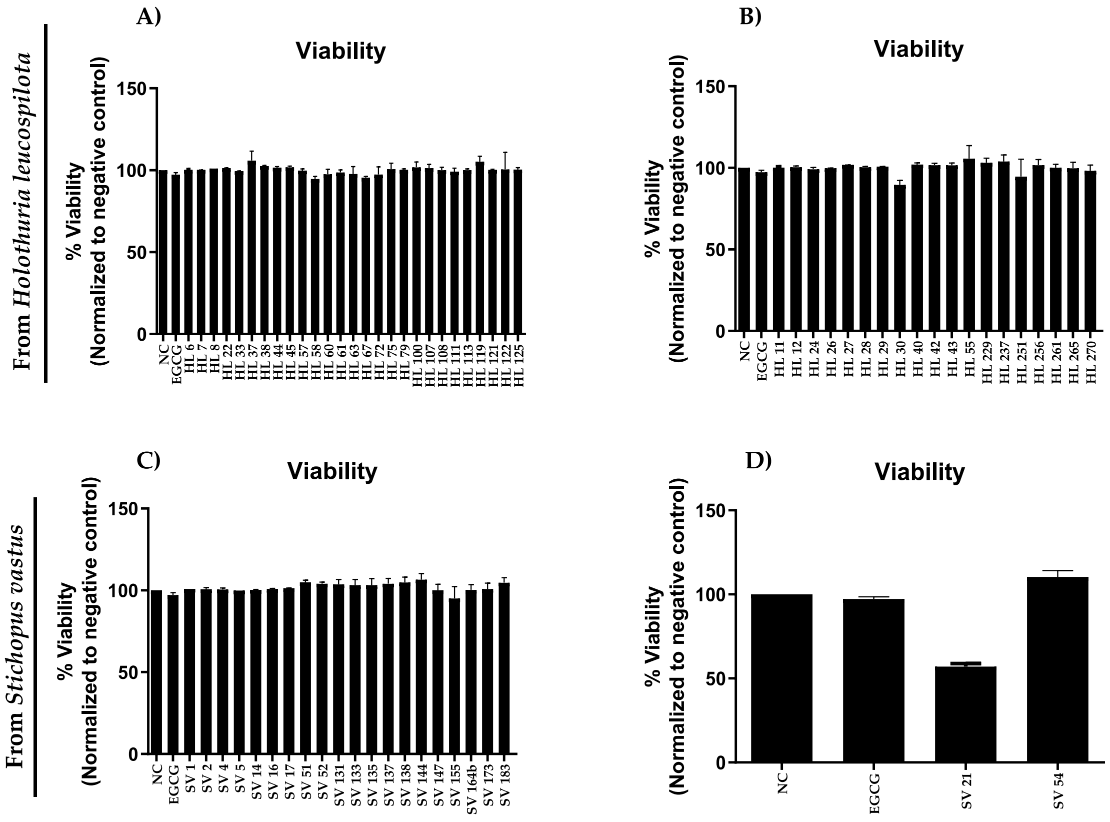

Figure S4. Viability of the liver cells in anti-HCV assay (NC – Negative control. EGCG - Epigallocatechin gallate.)

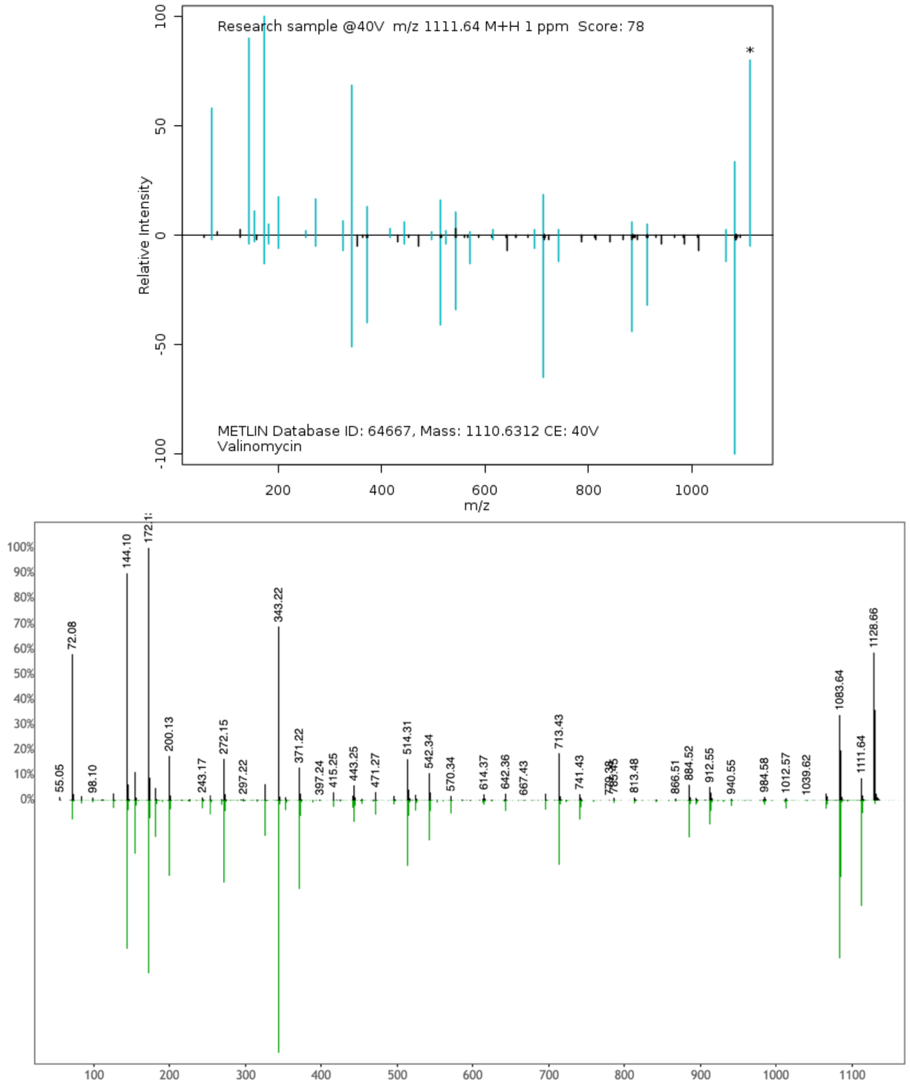

Figure S5. Identification of precursor from *Streptomyces cavourensis* SV 21 with *m/z* 1128.637 $[M+NH_4]^+$. MS^2 analysis of product ions in Metlin database library showed shared peaks with blue color. The score was 78 in collision energy 40 eV. In addition, mirror match sample (with MASST GNPS library (green lines) showed 60 shared peaks with valinomycin (precursor *m/z* 1111.64 $[M+H]^+$). Cosine score was 0.80.

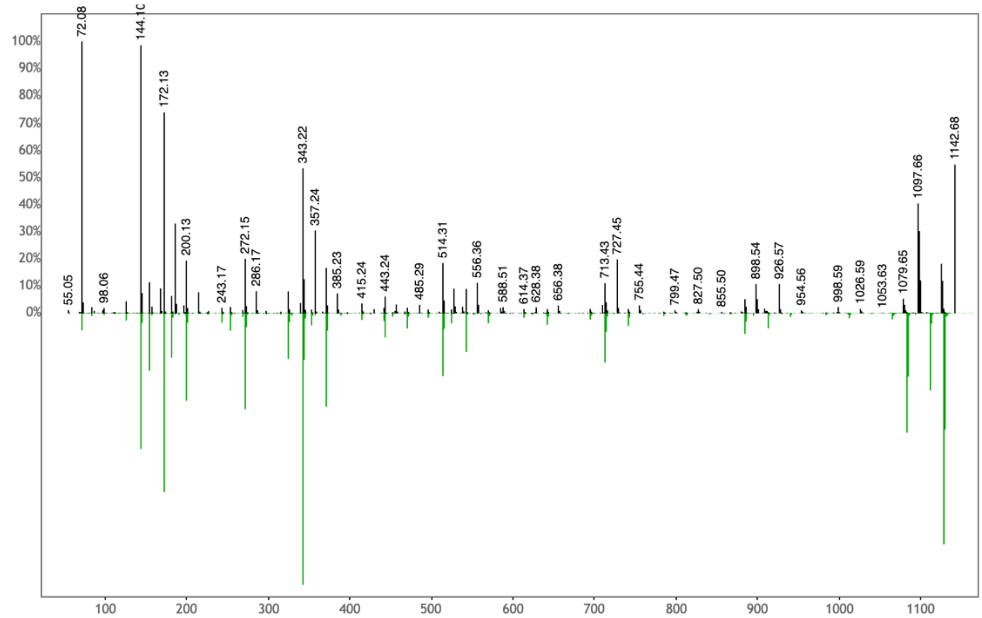

Figure S6. Identification of precursor from *Streptomyces cavourensis* SV 21 with *m/z* 1142.678 $[M+NH_4]^+$. Mirror match sample (black lines) with MASST GNPS library (green lines) showed 46 shared peaks with valinomycin (precursor *m/z* 1128.66 $[M+NH_4]^+$). Cosine score was 0.82. Delta of precursors *m/z* was 14.02.

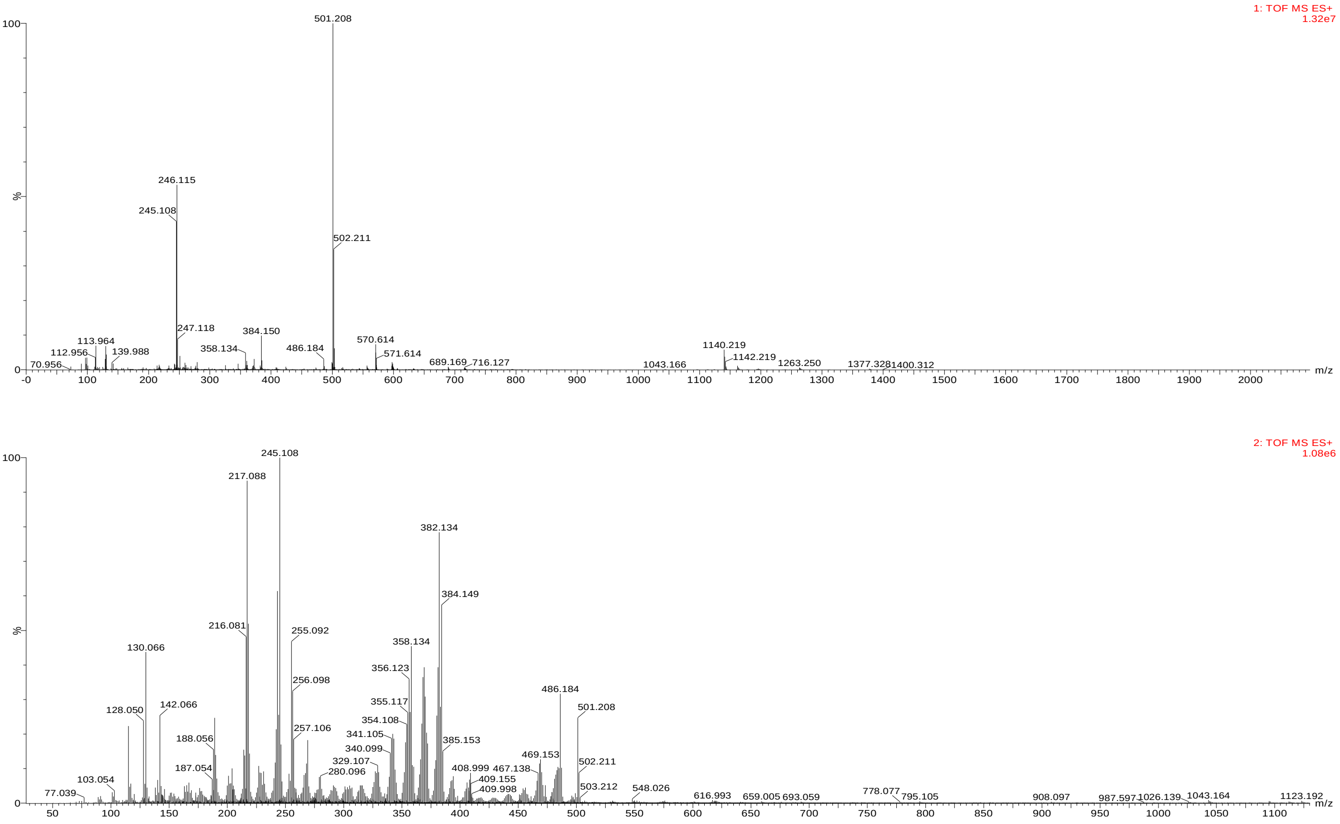

Figure S7. MS^1 and MS^2 spectra of precursor in *Kocuria flava* HL 55 with *m/z* 1140.219 $[M+H]^+$ which has no match either in MS^1 or MS^2 databases.

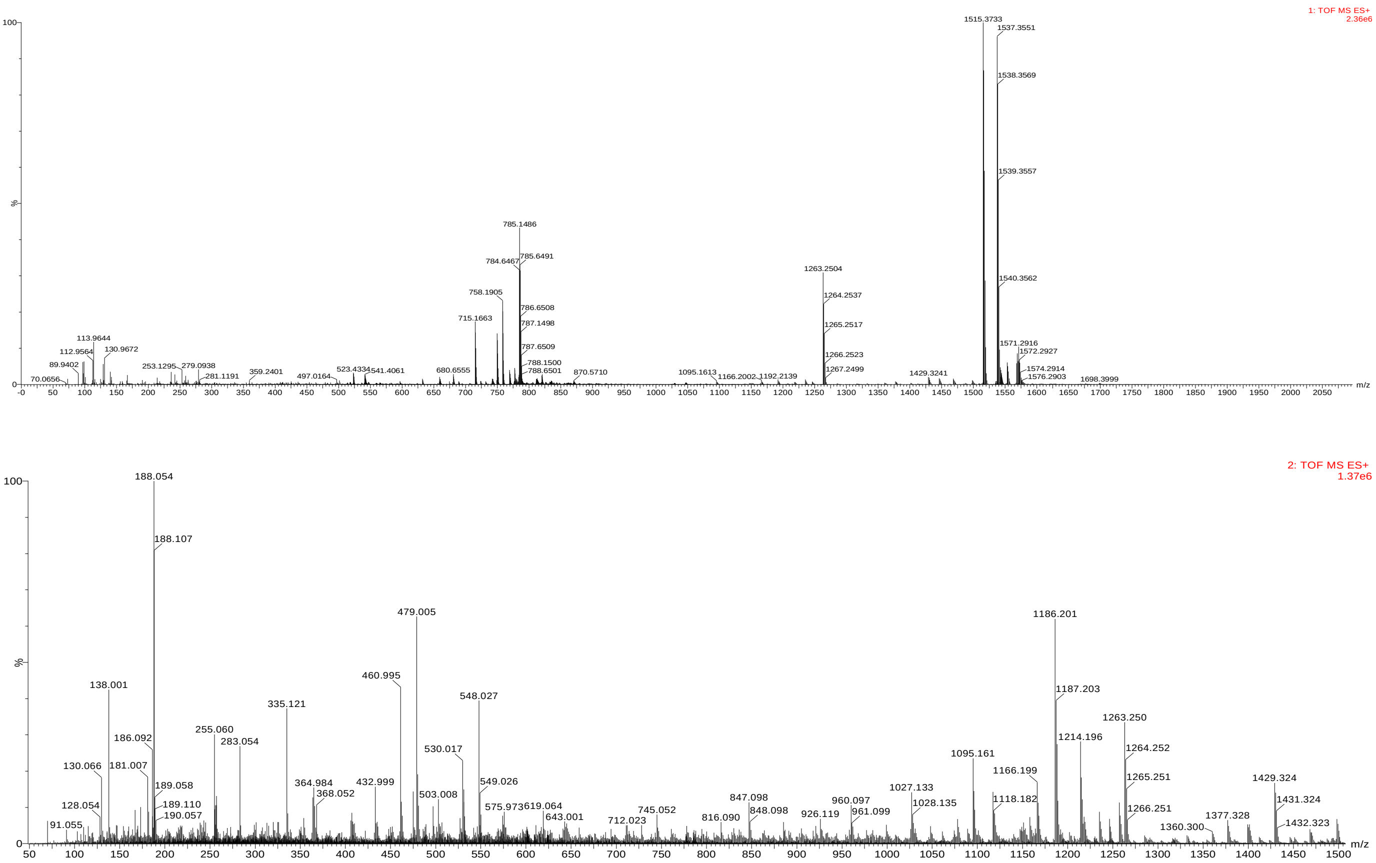

Figure S8. MS¹ and MS² spectra of precursor in *Kocuria flava* HL 55 with *m/z* 1515.373 $[M+H]^+$ which match with kocurin based on shared product ions with literature.

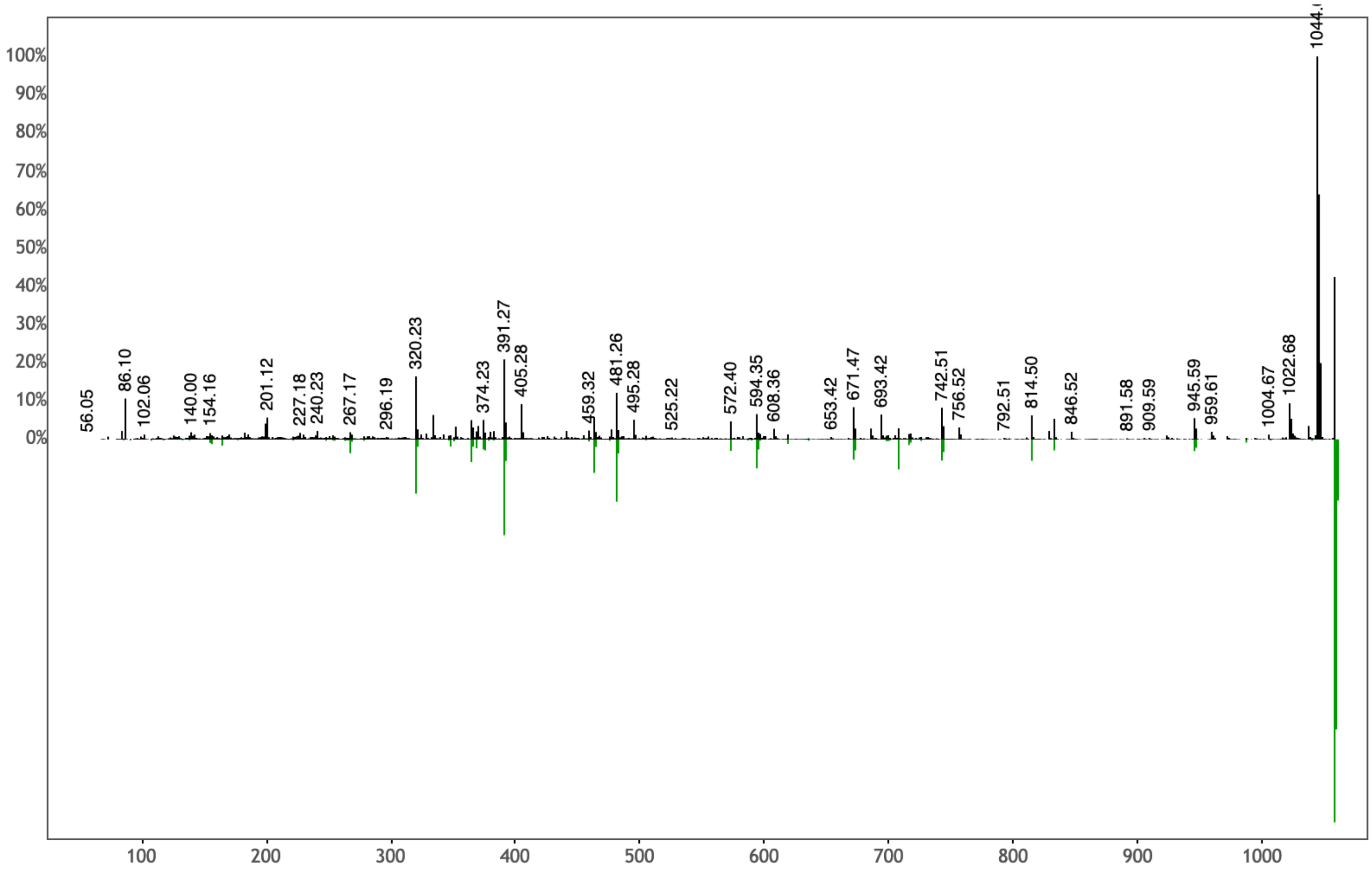

Figure S9. Identification of precursor from *Bacillus safensis* HL 63 and *Staphylococcus cohnii* subsp. *urealyticus* HL 67 with *m/z* 1058.671 [M+Na]$^+$. Mirror match sample (black lines) with MASST GNPS library (green lines) showed 26 shared peaks with surfactin (precursor *m/z* 1058.68 [M+Na]$^+$). Cosine score was 0.67. Delta of precursors m/z was 0.01.

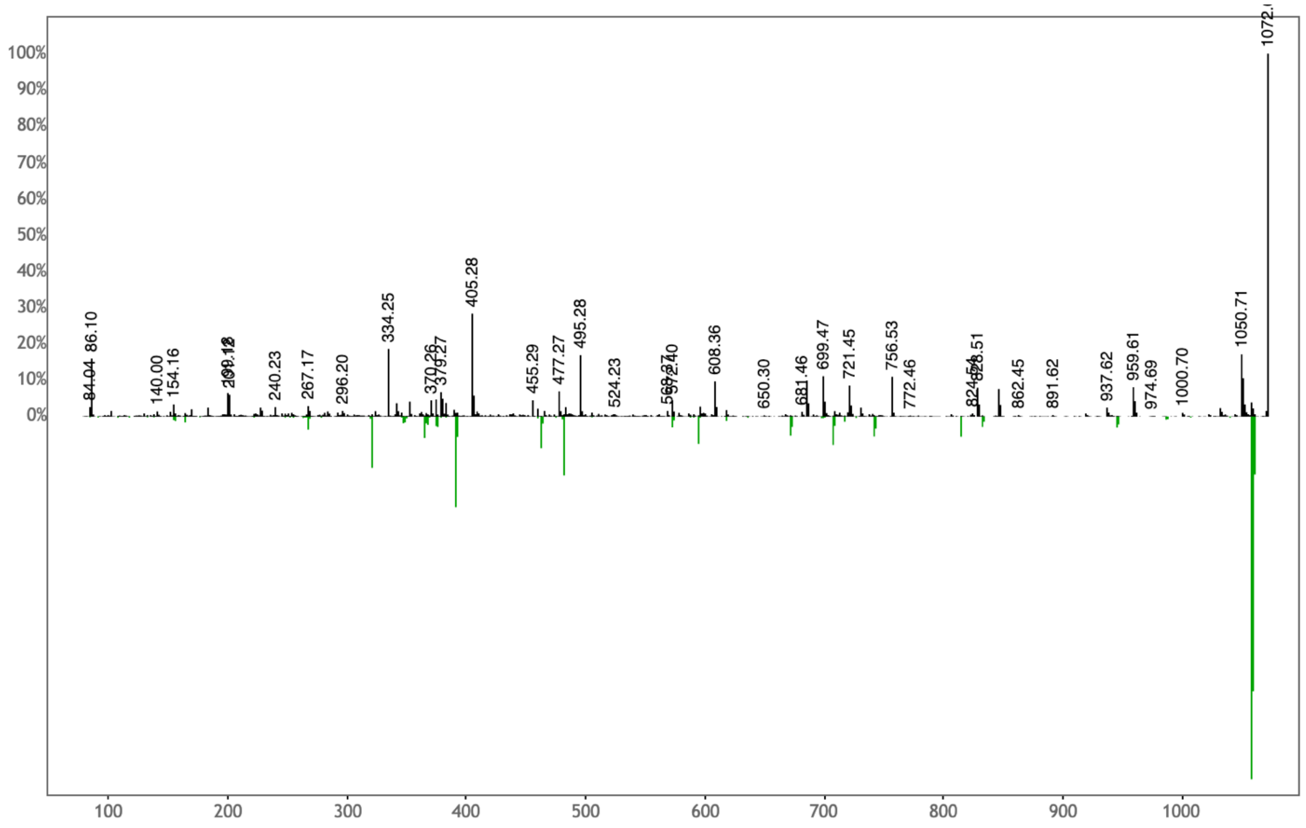

Figure S10. Identification of precursor from *Bacillus safensis* HL. 63 and *Staphylococcus cohnii* subsp. *urealyticus* HL 67 with *m/z* 1072.686 [M+Na]$^+$. Mirror match sample (black lines) with MASST GNPS library (green lines) showed 29 shared peaks with surfactin (precursor *m/z* 1058.67 [M+Na]$^+$). Cosine score was 0.73. Delta of precursors m/z was 14.02.

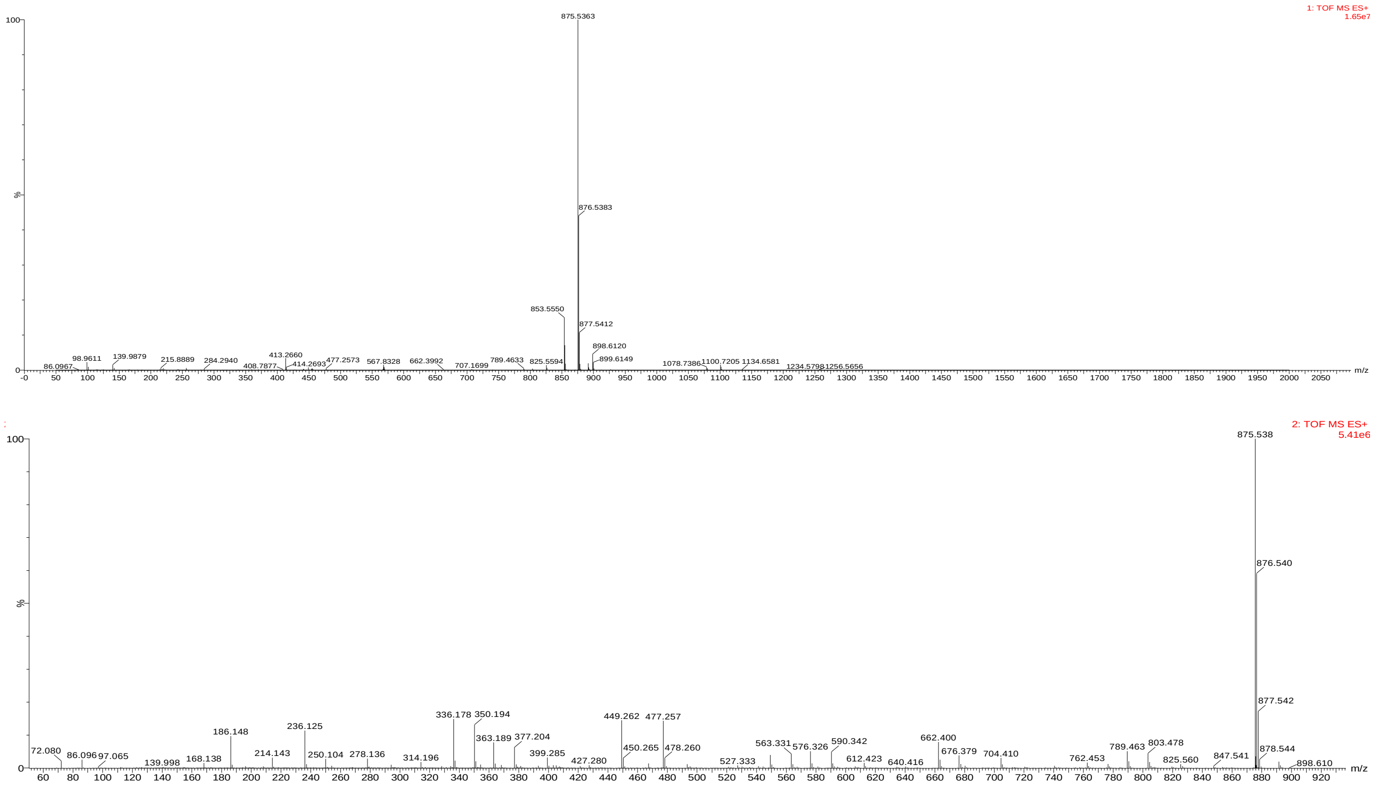

Figure S11. MS[1] and MS[2] spectra of precursor in *Bacillus safensis HL 63* with *m/z* 875.538 $[M+Na]^+$ which unidentified in MS[1] and MS[2] databases.

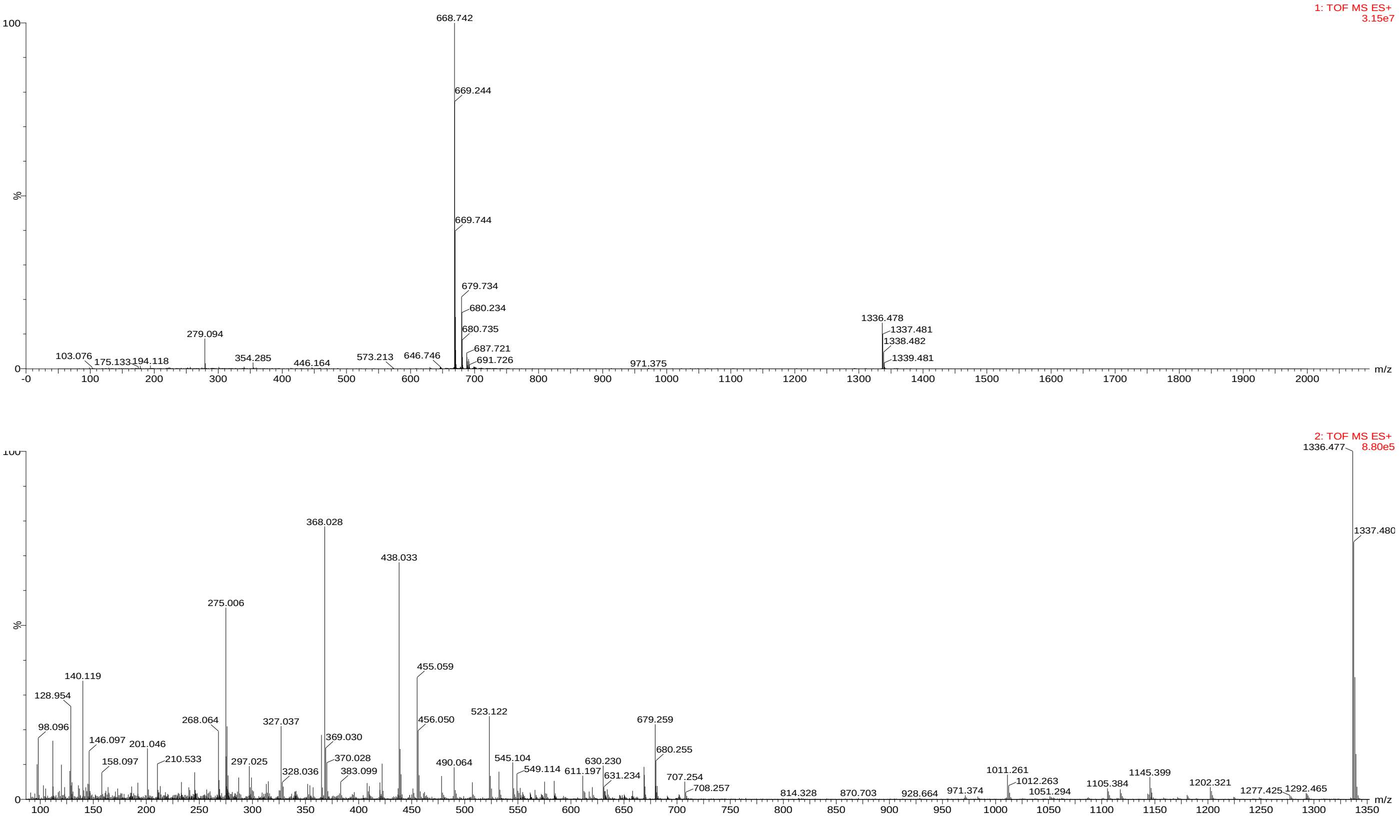

Figure S12. MS^1 and MS^2 spectra of precursor in *Bacillus safensis* SV. 147, *Paracoccus beibuensis* SV. 155, and *Nocardioides exalbidus* HL. 111 with *m/z* 1336.478 $[M+H]^+$ that matched with plantazolicin A based on shared product ions with literature.

Table S1. Total bacteria isolated from *Holothuria leucocpilota* (HL) and *Stichopus vastus* (SV). Closest type strain based on the NCBI database, accession and strain number, % similarity to the closest type strain, sequence length of the 16S rRNA-gene sequence, sample origin and antimicrobial activity are provided. The letter in parentheses in the antimicrobial column indicates the level of activity based on the last active location in the test-well (A-H) in 1:2 serial dilutions.

No.	Next related type strain	Sample accession Number	Type strain accession number	Similarity to type strain (%)	Sequence length (bp)	Sample Name	Antimicrobial Activity	
							Antibacteria	Antifungal
1.	*Acinetobacter schindleri*	MK696475	NR_025412.1	98.95	1047	HL 265 (int)	-	-
2.	*Bacillus aryabhattai*	MK696496	NR_115953.1	99.91	1134	HL 270 (int)	-	-
3.	*Bacillus cereus*	MK696514	NR_157734.1	99.91	1132	HL 229 (int)	-	-
4.	*Bacillus idriensis*	MK696468	NR_043268.1	99.47	948	HL 251 (int)	-	-
5.	*Bacillus safensis*	MK696463	NR_041794.1	100.00	927	SV 147 (ext)	Bs (B), Sa (H)	-
6.	*Bacillus safensis*	MK696525	NR_113945.1	99.91	1126	HL 63 (ext)	Sa (H)	-
7.	New genus of family Bacillaceae *(Bacillus sonorensis)**	MK696542	NR_113993.1	**92.88**	1081	HL 79 (ext)	Sa (A)	-
8.	*Brevibacterium luteolum*	MK696423	NR_114872.1	99.63	1076	SV 4 (ext)	-	-
9.	*Cellulosimicrobium funkei*	MK696437	NR_042937.1	99.78	915	HL 61 (ext)	-	-
10.	*Corynebacterium pilbarense*	MK696498	NR_116953.1	98.74	829	HL 119 (ext)	-	-
11.	*Dermacoccus nishinomiyaensis*	MK696488	NR_044872.1	99.72	1063	HL 57 (ext)	Bs (B), Sa (C)	Rg (A), Mh (A)
12.	*Dermacoccus profundi*	MK696484	NR_043262.1	99.89	1076	HL 11 (int)	-	-
13.	*Dermacoccus profundi*	MK696494	NR_043262.1	99.72	916	SV 127 (ext)	-	-
14.	*Dietzia maris*	MK696467	NR_118596.1	98.84	1126	SV 164b (ext)	Sa (A)	-
15.	*Epibacterium mobile*	MK696445	NR_114024.1	99.64	1112	HL 38 (ext)	Bs (B)	-
16.	*Erythrobacter vulgaris*	MK696434	NR_043136.1	99.18	980	SV 54 (int)	Ec (A)	-
17.	*Erythrobacter vulgaris*	MK696478	NR_043136.1	99.36	937	HL 45 (ext)	Bs (A)	-
18.	*Glutamicibacter* sp.* (*G. nicotianae*)	MK696438	NR_026190.1	**96.38**	1056	HL 108 (ext)	Bs (B), Sa (A)	Rg (A)
19.	*Isoptericola chiayiensis*	MK696432	NR_116696.1	98.88	894	HL 44 (ext)	Bs (A)	-
20.	*Isoptericola chiayiensis*	MK696541	NR_116696.1	98.98	885	HL 74 (ext)	NT	NT
21.	*Janibacter alkaliphilus*	MK696433	NR_109453.1	98.92	1018	SV 51 (ext)	Bs (A)	-
22.	*Janibacter anophelis*	MK696442	NR_043218.1	99.15	1062	HL 24 (int)	-	-
23.	*Janibacter melonis*	MK696486	NR_025805.1	99.79	964	HL 40 (int)	Bs (A)	-
24.	*Kocuria flava*	MK696544	NR_044308.1	99.21	892	HL 55 (int)	Bs(E), Ec (A), Sa (D)	-
25.	*Kocuria palustris*	MK696435	NR_026451.1	99.90	1045	HL 6 (ext)	Bs (C), Sa (C)	Rg (A)
26.	*Kocuria palustris*	MK696424	NR_026451.1	100.00	956	HL 7 (ext)	Bs (B)	-
27.	*Kocuria palustris*	MK696425	NR_026451.1	100.00	922	HL 8 (ext)	Bs (A)	-
28.	*Kocuria palustris*	MK696524	NR_026451.1	99.81	879	HL 60 (ext)	-	-
29.	*Kocuria palustris*	MK696492	NR_026451.1	100.00	827	HL 65 (ext)	NT	NT
30.	*Kocuria* sp.* (*K. palustris*)	MK696490	NR_026451.1	**97.64**	982	HL 66 (ext)	NT	NT
31.	*Kocuria palustris*	MK696426	NR_026451.1	100.00	1021	HL 12 (int)	Bs (B)	-
32.	*Kocuria palustris*	MK696521	NR_026451.1	100.00	1048	HL 31 (int)	NT	NT
33.	*Kocuria palustris*	MK696522	NR_026451.1	99.71	917	HL 42 (int)	-	-
34.	*Kocuria palustris*	MK696516	NR_026451.1	99.81	1044	HL 234 (int)	NT	NT
35.	*Kocuria palustris*	MK696537	NR_026451.1	99.73	972	HL 245 (int)	NT	NT
36.	*Kocuria* sp.* (*K. palustris*)	MK720778	NR_026451.1	**97.45**	1063	HL 268 (int)	NT	NT
37.	*Kocuria palustris*	MK720779	NR_026451.1	98.63	967	HL 273 (int)	NT	NT

38.	*Kocuria palustris*	MK696441	NR_026451.1	98.76	913	SV 14 (ext)	-	-
39.	*Kocuria palustris*	MK696485	NR_026451.1	98.01	1045	SV 15a (ext)	NT	NT
40.	*Kocuria palustris*	MK696538	NR_026451.1	99.78	1071	SV 15b (ext)	NT	NT
41.	*Kocuria palustris*	MK696481	NR_026451.1	99.77	737	SV 146 (ext)	NT	NT
42.	*Kocuria palustris*	MK696465	NR_026451.1	99.76	826	SV 156 (ext)	NT	NT
43.	*Kocuria palustris*	MK696534	NR_026451.1	99.59	877	SV 195 (int)	NT	NT
44.	*Kytococcus sedentarius*	MK696450	NR_074714.2	99.61	1062	HL 109 (ext)	NT	NT
45.	*Kytococcus sedentarius*	MK696453	NR_074714.2	99.71	1017	HL 115 (ext)	NT	NT
46.	*Kytococcus sedentarius*	MK696431	NR_074714.2	99.88	1041	HL 30 (int)	Bs (A)	-
47.	*Kytococcus sedentarius*	MK696446	NR_074714.2	99.72	838	HL 43 (int)	-	-
48.	*Kytococcus sedentarius*	MK696469	NR_074714.2	99.59	1054	HL 252 (int)	NT	NT
49.	*Kytococcus sedentarius*	MK696470	NR_074714.2	99.22	964	HL 253 (int)	NT	NT
50.	*Kytococcus sedentarius*	MK696471	NR_074714.2	98.93	1026	HL 254 (int)	NT	NT
51.	*Kytococcus* sp.* (*K. sedentarius*)	MK720780	NR_074714.2	**97.58**	942	HL 255 (int)	NT	NT
52.	*Kytococcus sedentarius*	MK696474	NR_074714.2	99.38	953	HL 262 (int)	NT	NT
53.	*Kytococcus sedentarius*	MK696483	NR_074714.2	99.72	980	SV 2 (ext)	Bs (B)	-
54.	*Leucobacter tardus*	MK696518	NR_042694.1	99.23	1172	HL 259 (int)	NT	NT
55.	*Mariniluteicoccus* sp.* (*M. endophyticus*)	MK696508	NR_148778.1	**96.26**	1042	SV 203 (int)	NT	NT
56.	*Micrococcus aloeverae*	MK696444	NR_134088.1	99.78	1041	HL 33 (ext)	-	-
57.	*Micrococcus aloeverae*	MK696430	NR_134088.1	99.79	937	HL 29 (int)	Bs (A)	-
58.	*Micrococcus aloeverae*	MK696436	NR_134088.1	99.52	918	SV 5 (ext)	Bs (A)	Rg (A)
59.	*Micrococcus aloeverae*	MK696523	NR_134088.1	99.36	908	SV 52 (ext)	-	-
60.	*Micrococcus aloeverae*	MK696460	NR_134088.1	99.78	959	SV 136 (ext)	NT	NT
61.	*Micrococcus endophyticus*	MK696473	NR_044365.1	98.62	1018	HL 261 (int)	-	-
62.	*Micrococcus flavus*	MK696517	NR_043881.1	99.20	1005	HL 237 (int)	Bs (B), Sa (B), Rg (B)	-
63.	*Micrococcus terreus*	MK696528	NR_116649.1	99.44	1081	SV 137 (ext)	-	-
64.	*Micrococcus yunnanensis*	MK696527	NR_116578.1	99.35	1071	HL 116 (ext)	NT	NT
65.	*Micrococcus yunnanensis*	MK696443	NR_116578.1	98.81	923	HL 32 (int)	NT	NT
66.	*Nocardioides* sp.* (*N. exalbidus*)	MK696451	NR_041526.1	**97.96**	1036	HL 111 (ext)	Bs (A), Sa (H)	-
67.	*Nocardioides exalbidus*	MK696464	NR_041526.1	98.55	1076	SV 152 (ext)	NT	NT
68.	*Ornithinimicrobium kibberense*	MK696459	NR_043056.1	99.59	988	SV 135 (ext)	-	-
69.	*Pantoea septica*	MK696487	NR_116752.1	99.14	1080	SV 138 (ext)	Bs (A)	-
70.	New genus of family Rhodobacteraceae (*Paracoccus beibuensis*)	-	NR_116400.1	**93.08**	1011	SV 155 (ext)	Sa (E)	-
71.	*Paracoccus* sp.* (*P. koreensis*)	MK696429	NR_114060.1	**97.33**	940	HL 28 (int)	Bs (A)	Mh (A)
72.	*Paracoccus marinus*	MK696491	NR_113921.1	99.03	928	HL 256 (int)	Bs (A), Sa (A)	-
73.	*Paracoccus sulfuroxidans*	MK696428	NR_043887.1	98.25	861	HL 27 (int)	-	-
74.	*Pseudoalteromonas shioyasakiensis*	MK696502	NR_125458.1	99.38	1127	SV 163 (ext)	NT	NT
75.	*Pseudomonas stutzeri*	MK696497	NR_041715.1	99.21	1079	HL 26 (int)	Bs (B)	-
76.	*Pseudomonas stutzeri*	MK696495	NR_041715.1	99.60	1013	HL 231 (int)	NT	NT
77.	*Pseudomonas stutzeri*	MK696493	NR_041715.1	99.72	1010	SV 128 (ext)	NT	NT
78.	New Genus of family Propionibacteriaceae* (*Pseudopropionibacterium profundi*)	MK696480	NR_159102.1	**93.29**	1047	SV 17 (ext)	-	-
79.	*Psychrobacter celer*	MK696489	NR_043225.1	99.27	1100	HL 58 (ext)	-	-
80.	*Psychrobacter marincola*	MK696539	NR_025458.1	99.40	1165	HL 72 (ext)	Sa (A)	-
81.	*Rothia kristinae*	MK696476	NR_026199.1	99.53	1059	HL 36 (ext)	NT	NT
82.	*Rothia kristinae*	MK696477	NR_026199.1	99.29	989	HL 37 (ext)	-	-

83.	*Serinicoccus* sp.* (*S. profundi*)	MK696482	NR_116387.1	**97.91**	719	SV 16 (ext)	-	-
84.	*Staphylococcus arlettae*	MK696500	NR_024664.1	99.65	1149	SV 133 (ext)	-	-
85.	*Staphylococcus arlettae*	MK696504	NR_024664.1	100.00	1110	SV 165 (ext)	NT	NT
86.	*Staphylococcus arlettae*	MK696533	NR_024664.1	99.55	1126	SV 184 (ext)	NT	NT
87.	*Staphylococcus cohnii*	MK696452	NR_036902.1	99.80	990	HL 113 (ext)	-	-
88.	*Staphylococcus cohnii* subsp. *urealyticus*	MK696447	NR_037046.1	99.31	1121	HL 67 (ext)	Sa (H)	-
89.	*Staphylococcus cohnii* subsp. *urealyticus*	MK696448	NR_037046.1	99.36	986	HL 68 (ext)	NT	NT
90.	*Staphylococcus cohnii* subsp. *urealyticus*	MK696540	NR_037046.1	100.00	1098	HL 73 (ext)	NT	NT
91.	*Staphylococcus cohnii* subsp. *urealyticus*	MK696535	NR_037046.1	100.00	1084	HL 232 (int)	NT	NT
92.	*Staphylococcus cohnii* subsp. *urealyticus*	MK696536	NR_037046.1	99.82	1010	HL 235 (int)	NT	NT
93.	*Staphylococcus cohnii* subsp. *urealyticus*	MK696422	NR_037046.1	100.00	1116	SV 1 (ext)	Bs (B)	-
94.	*Staphylococcus cohnii* subsp. *urealyticus*	MK696457	NR_037046.1	100.00	1115	SV 130 (ext)	NT	NT
95.	*Staphylococcus cohnii* subsp. *urealyticus*	MK696458	NR_037046.1	99.73	953	SV 131 (ext)	-	-
96.	*Staphylococcus cohnii* subsp. *urealyticus*	MK696462	NR_037046.1	99.45	1009	SV 143 (ext)	NT	NT
97.	*Staphylococcus cohnii* subsp. *urealyticus*	MK696440	NR_037046.1	99.80	1098	SV 144 (ext)	Sa (A)	-
98.	*Staphylococcus cohnii* subsp. *urealyticus*	MK696529	NR_037046.1	99.64	1111	SV 169 (ext)	NT	NT
99.	*Staphylococcus cohnii* subsp. *urealyticus*	MK696530	NR_037046.1	99.91	1076	SV 170 (ext)	NT	NT
100.	*Staphylococcus cohnii* subsp. *urealyticus*	MK696510	NR_037046.1	99.90	1082	SV 208 (int)	NT	NT
101.	*Staphylococcus edaphicus*	MK696526	NR_156818.1	99.73	1108	HL 75 (ext)	Bs (C), Sa (B)	Rg (B)
102.	*Staphylococcus haemolyticus*	MK696532	NR_113345.1	99.33	1044	SV 183 (ext)	-	-
103.	*Staphylococcus haemolyticus*	MK696509	NR_113345.1	99.82	1109	SV 204 (int)	NT	NT
104.	*Staphylococcus haemolyticus*	MK696511	NR_113345.1	99.55	1121	SV 212 (int)	NT	NT
105.	*Staphylococcus haemolyticus*	MK696512	NR_113345.1	99.89	914	SV 214 (int)	NT	NT
106.	*Staphylococcus hominis* subsp. *novobiosepticus*	MK696506	NR_041323.1	99.55	1119	SV 199 (int)	NT	NT
107.	*Staphylococcus hominis* subsp. *novobiosepticus*	MK696507	NR_041323.1	99.91	1113	SV 202 (int)	NT	NT
108.	*Staphylococcus pasteuri*	MK696505	NR_114435.1	99.74	1163	SV 166 (ext)	NT	NT
109.	*Staphylococcus pasteuri*	MK696531	NR_114435.1	99.91	1117	SV 173 (ext)	-	-
110.	*Staphylococcus warneri*	MK696543	NR_025922.1	99.91	1084	HL 100 (ext)	Sa (A)	-
111.	*Streptomyces cavourensis*	MK696479	NR_043851.1	100.00	1034	SV 21 (int)	Bs (H), Sa (E)	Mh (G)
112.	*Tenacibaculum lutimaris*	MK696503	NR_043080.1	99.82	1109	SV 164c (ext)	NT	NT
113.	*Vibrio alginolyticus*	MK696427	NR_118258.1	99.52	1039	HL 22 (ext)	Bs (G), Sa (E), Ms (A)	Rg (B), Mh (B)
114.	*Vibrio alginolyticus*	MK696455	NR_118258.1	99.90	1083	HL 123 (ext)	NT	NT
115.	*Vibrio alginolyticus*	MK696472	NR_118258.1	98.42	1094	HL 258 (int)	NT	NT
116.	*Vibrio alginolyticus*	MK696519	NR_118258.1	99.18	841	HL 272 (int)	NT	NT
117.	*Vibrio alginolyticus*	MK696461	NR_118258.1	99.81	964	SV 140 (ext)	NT	NT
118.	*Vibrio alginolyticus*	MK696439	NR_118258.1	99.63	1012	SV 141 (ext)	NT	NT
119.	*Vibrio alginolyticus*	MK696466	NR_118258.1	98.72	1091	SV 158 (ext)	NT	NT
120.	*Vibrio alginolyticus*	MK696501	NR_122059.1	98.98	1178	SV 159 (ext)	NT	NT
121.	*Vibrio* sp.* (*V. harveyi*)	MK696456	NR_043165.1	**96.23**	1074	HL 125 (ext)	Bs (A), Sa (A)	-
122.	*Vibrio harveyi*	MK696454	NR_113784.1	99.80	996	HL 121 (ext)	Sa (A)	Rg (A)
123.	*Vibrio owensii*	MK696449	NR_117424.1	99.34	907	HL 107 (ext)	-	-
124.	*Vibrio owensii*	MK696499	NR_117424.1	99.44	1082	HL 122 (ext)	Sa (A)	Rg (A)
125.	*Vibrio owensii*	MK696513	NR_117424.1	99.46	1105	HL 226 (int)	NT	NT
126.	*Vibrio owensii*	MK696515	NR_117424.1	98.99	1087	HL 230 (int)	NT	NT
127.	*Vibrio owensii*	MK696520	NR_117424.1	99.55	1106	HL 274 (int)	NT	NT

(*): represent new bacterial species (closest match in NCBI database). Bs: *Bacillus subtilis*, Ec: *Escherichia coli*, Mh: *Mucor hiemalis*, Rg: *Rhodotorula glutinis*, Sa: *Staphylococcus aureus*; HL: *Holothuria leucocpilota* ; SV: *Stichopus vastus* ; - : not active ; NT: Not tested; (int): isolated from internal part, (ext): isolated from external part.

Table S2. Search results of the detected precursor ions and its exact masses in databases namely MarinLit, Dictionary of Natural Products (DNP), Global Natural Products Social Molecular Networking (GNPS), and Metlin.

Strain Bacteria	M (in Da) And RT (in min)	Precursor ions (m/z)	Putatively Matching Compound from Database/Library (M±0.01-0.005)			MS1 Databases			
			Substance (s) or compound (s)	M	Molecular formula	MarinLit	DNP	GNPS	Metlin
Streptomyces cavourensis. SV 21	457.174 (5.34)	458.181 $[M+H]^+$	Chryscandin	457.171	$C_{20}H_{23}N_7O_6$	-	Y	-	-
			Dankastatin B	457.179	$C_{23}H_{33}Cl_2NO_4$	Y	-	-	-
			Gymnastatin R	457.179	$C_{23}H_{33}Cl_2NO_4$	Y	-	-	-
			Insulicolide C	457.174	$C_{24}H_{27}NO_8$	Y	-	-	-
			Medermycin	457.174	$C_{24}H_{27}NO_8$	-	Y	-	Y
			5-Methyldihydrofolic acid	457.171	$C_{20}H_{23}N_7O_6$	-	-	-	Y
			5,10-Methylenetetrahydrofolate	457.171	$C_{20}H_{23}N_7O_6$	-	-	-	Y
	489.200 (5.93)	490.207 $[M+H]^+$	Altemicidin; 5'-N-(2S-Amino-3S-methylpentanoyl)	489.1893	$C_{19}H_{31}N_5O_8S$	-	Y	-	-
			Aniquinazoline C	489.2012	$C_{26}H_{27}N_5O_5$	Y	-	-	-
			Antibiotic OA 6129E	489.2145	$C_{21}H_{35}N_3O_8S$	-	Y	-	Y
			Fiscalin A; 1β-Hydroxy	489.2012	$C_{26}H_{27}N_5O_5$	-	Y	-	-
			Haouamine A	489.1940	$C_{32}H_{27}NO_4$	Y	Y	-	-
			Hetisan-2,11,13,15-tetrol; (11α,13R,15β)-form, 2-Ketone, 11-benzoyl, 15-Ac	489.2151	$C_{29}H_{31}NO_6$	-	Y	-	-
			Ryanodine; 8-Oxo, 10-deoxy, 9,10-didehydro	489.1999	$C_{25}H_{31}NO_9$	-	Y	-	-
			Terbinafine metabolite glucuronide	489.1999	$C_{25}H_{31}NO_9$	-	-	-	Y
	1110.630 (19.24)	1128.665 $[M+NH4]^+$	Valinomycin	1110.6311	$C_{54}H_{90}N_6O_{18}$	Y	-	Y	Y
	1124.644 (20.08)	1142.678 $[M+NH4]^+$	UNIDENTIFIED	-	-	-	-	-	-
	662.447 (22.06)	663.454 $[M+H]^+$	3-hexanoyl-NBD Cholesterol	662.4407	$C_{39}H_{58}N_4O_5$	-	-	-	Y
			Eryloside T	662.4394	$C_{38}H_{62}O_9$	Y	-	-	-
			Goyaglycoside c	662.4394	$C_{38}H_{62}O_9$	-	-	-	Y
Kocuria flava HL 55	1139.211 (10.67)	1140.219 $[M+H]^+$	UNIDENTIFIED	-	-	-	-	-	-
	1514.366 (10.94)	1515.373 $[M+H]^+$	Baringolin	1514.3660	$C_{69}H_{66}O_{13}N_{18}S_5$	Y	-	-	-
			Kocurin	1514.3660	$C_{69}H_{66}N_{18}O_{13}S_5$	Y	Y	-	-

Bacillus safensis HL 63 and *Staphylococcus cohnii* subsp. *urealyticus* HL 67	1069.636 (14.77)	1070.643 $[M+H]^+$	UNIDENTIFIED	-	-	-	-	-	-
	1101.609 (14.65)	1102.616 $[M+H]^+$	Callitachykinin II	1101.6043	$C_{48}H_{79}N_{17}O_{13}$	-	Y	-	-
	1053.640 (16.06)	1076.629 $[M+Na]^+$	UNIDENTIFIED	-	-	-	-	-	-
	1067.654 (18.23)	1068.661 $[M+H]^+$	Azalomycin F3a	1067.6504	$C_{55}H_{93}N_3O_{17}$	Y	-	-	-
			Azalomycin F; Azalomycin F3	1067.6505	$C_{55}H_{93}N_3O_{17}$	-	Y	-	-
			Azalomycin F; Azalomycin F3, 2-Demethyl, N-Me	1067.6505	$C_{55}H_{93}N_3O_{17}$	-	Y	-	-
	1021.667 (18.35)	1022.674 $[M+H]^+$	Antibiotic BO 7: Antibiotic BO 7-1	1021.6675	$C_{52}H_{91}N_7O_{13}$	-	Y	-	-
			Bacillus amyloliquefaciens surfactin 2	1021.6675	$C_{52}H_{91}N_7O_{13}$	-	Y	-	-
			Bacircines; Bacircine 2	1021.6675	$C_{52}H_{91}N_7O_{13}$	-	Y	-	-
			Bacircines; Bacircine 3	1021.6675	$C_{52}H_{91}N_7O_{13}$	-	Y	-	-
			Gageopectin B	1021.6675	$C_{52}H_{91}N_7O_{13}$	Y	-	-	-
			Surfactin	1021.66	$C_{52}H_{91}N_7O_{14}$	-	-	Y	-
			Surfactin; ai-C14 surfactin	1021.6675	$C_{52}H_{91}N_7O_{13}$	-	Y	-	-
			Surfactin; surfactin B1	1021.6675	$C_{52}H_{91}N_7O_{13}$	-	Y	-	-
			Surfactin; surfactin B2	1021.6675	$C_{52}H_{91}N_7O_{13}$	-	Y	-	-
	1035.684 (18.46)	1058.671 $[M+Na]^+$	$C_{53}H_{93}N_7O_{13}$	1035.683	$C_{53}H_{93}N_7O_{13}$	Y	-	-	-
			Antibiotic BO 7: Antibiotic BO 7-2	1035.684	$C_{53}H_{93}N_7O_{13}$	-	Y	-	-
			Bacillus amyloliquefaciens surfactin 1	1035.684	$C_{53}H_{93}N_7O_{13}$	-	Y	-	-
			Bacillus pumilus KMM 1364 Lipodepsipeptides; KMM1364A	1035.684	$C_{53}H_{93}N_7O_{13}$	-	Y	-	-
			Bacillus pumilus KMM 1364 Lipodepsipeptides; KMM1364B	1035.684	$C_{53}H_{93}N_7O_{13}$	-	Y	-	-
			Bacircines; Bacircine 4	1035.684	$C_{53}H_{93}N_7O_{13}$	-	Y	-	-
			Bacircines; Bacircine 5	1035.684	$C_{53}H_{93}N_7O_{13}$	-	Y	-	-
			Daitocidin; Daitocidin A1	1035.684	$C_{53}H_{93}N_7O_{13}$	-	Y	-	-
			Gageopeptin A	1035.683	$C_{53}H_{93}N_7O_{13}$	Y	-	-	-
			Pumilacidin B	1035.683	$C_{53}H_{93}N_7O_{13}$	Y	-	-	-
			Surfactin	1035.683	$C_{53}H_{93}N_7O_{13}$	-	-	-	Y
			Surfactin; C15 surfactin	1035.684	$C_{53}H_{93}N_7O_{13}$	-	Y	-	-
			Surfactin; surfactin C1	1035.684	$C_{53}H_{93}N_7O_{13}$	-	Y	-	-
			Surfactin; surfactin C2	1035.684	$C_{53}H_{93}N_7O_{13}$	-	Y	-	-
			Surfactin C	1035.680	$C_{53}H_{93}N_7O_{14}$	-	-	Y	-
	1049.698 (18.87)	1072.686 $[M+Na]^+$	Antibiotic BO 7: Antibiotic BO 7-3	1049.6988	$C_{54}H_{95}N_7O_{13}$	-	Y	-	-

			Bacillus pumilus KMM 1364 Lipodepsipeptides; KMM1364C	1049.6988	$C_{54}H_{95}N_7O_{13}$	-	Y	-	-
			Bacillus pumilus KMM 1364 Lipodepsipeptides; KMM1364D	1049.6988	$C_{54}H_{95}N_7O_{13}$	-	Y	-	-
			Daitocidin; Daitocidin B1	1049.6988	$C_{54}H_{95}N_7O_{13}$	-	Y	-	-
			Daitocidin; Pumilacidin F	1049.6988	$C_{54}H_{95}N_7O_{13}$	-	Y	-	-
			Daitocidin; Pumilacidin G	1049.6988	$C_{54}H_{95}N_7O_{13}$	-	Y	-	-
			Pumilacidin A	1049.6987	$C_{54}H_{95}N_7O_{13}$	Y	-	-	-
			Surfactin; ai-C16 surfactin	1049.6988	$C_{54}H_{95}N_7O_{13}$	-	Y	-	-
			Surfactin; surfactin D	1049.6988	$C_{54}H_{95}N_7O_{13}$	-	Y	-	-
	1095.685 (19.32)	1096.692 $[M+H]^+$	Azalomycin F5a	1095.6817	$C_{57}H_{97}N_3O_{17}$	Y	-	-	-
			$C_{57}H_{97}N_3O_{17}$	1095.6818	$C_{57}H_{97}N_3O_{17}$	Y	-	-	-
			Azalomycin F; Azalomycin F3, N,N-Di-Me	1095.6818	$C_{57}H_{97}N_3O_{17}$	-	Y	-	-
			Shurimycin A; N-Me	1095.6818	$C_{57}H_{97}N_3O_{17}$	-	Y	-	-
	1063.714 (19.48)	1086.702 $[M+Na]^+$	Pumilacidin D	1063.7144	$C_{55}H_{97}N_7O_{13}$	Y	-	-	-
			Pumilacidin E	1063.7144	$C_{55}H_{97}N_7O_{13}$	Y	-	-	-
	852.545 (19.72)	875.534 $[M+Na]^+$	UNIDENTIFIED	-	-	-	-	-	-
	1077.723 (19.8)	1100.717 $[M+Na]^+$	Pumilacidin C	1077.7300	$C_{56}H_{99}N_7O_{13}$	Y	-	-	-
Staphylococcus edaphicus HL 75	346.205 (13.6)	347.212 $[M+H]^+$	(3S,4S)-3-hydroxytetradecane-1,3,4-tricarboxylic acid	346.1992	$C_{17}H_{30}O_7$	-	-	-	Y
			[10]-Dehydrogingerdione	346.2144	$C_{21}H_{30}O_4$	-	-	-	Y
			corticosterone	346.2144	$C_{21}H_{30}O_4$	-	-	-	Y
	394.206 (15.06)	395.213 $[M+H]^+$	6β-acetoxyolepupuane	394.1991	$C_{21}H_{30}O_7$	Y	-	-	-
			$C_{21}H_{30}O_7$	394.1992	$C_{21}H_{30}O_7$	Y	-	-	-
			Curvulaide A	394.2100	$C_{25}H_{30}O_4$	Y	-	-	-
			2,8,14-triacetoxycapnell-9(12)-ene-10-ol	394.1991	$C_{21}H_{30}O_7$	Y	-	-	-
			Capnellene	394.1991	$C_{21}H_{30}O_7$	Y	-	-	-
			Nigakilactone N	394.1992	$C_{21}H_{30}O_7$	-	-	-	Y
			Pteroside Z	394.1992	$C_{21}H_{30}O_7$	-	-	-	Y
			Secoeremopetasitolide B	394.1992	$C_{21}H_{30}O_7$	-	-	-	Y
Bacillus safensis SV 147, *Paracoccus beibuensis* SV 155, and *Nocardioides exalbidus* HL 111	1335.471 (10.43)	1336.478 $[M+H]^+$	Plantazolicin	1335.4702	$C_{36}H_{69}N_{17}O_{13}S_2$	-	Y	-	-
	1021.668 (18.13)	1044.657 $[M+Na]^+$	Antibiotic BO 7: Antibiotic BO 7-1	1021.6675	$C_{52}H_{91}N_7O_{13}$	-	Y	-	-
			Bacillus amyloliquefaciens surfactin 2	1021.6675	$C_{52}H_{91}N_7O_{13}$	-	Y	-	-

			Bacircines; Bacircine 2	1021.6675	$C_{52}H_{91}N_7O_{13}$	-	Y	-	-
			Bacircines; Bacircine 3	1021.6675	$C_{52}H_{91}N_7O_{13}$	-	Y	-	-
			Gageopectin B	1021.6675	$C_{52}H_{91}N_7O_{13}$	Y	-	-	-
			Surfactin	1021.66	$C_{52}H_{91}N_7O_{14}$	-	-	Y	-
			Surfactin; ai-C14 surfactin	1021.6675	$C_{52}H_{91}N_7O_{13}$	-	Y	-	-
			Surfactin; surfactin B1	1021.6675	$C_{52}H_{91}N_7O_{13}$	-	Y	-	-
			Surfactin; surfactin B2	1021.6675	$C_{52}H_{91}N_7O_{13}$	-	Y	-	-
	1035.683 (18.2)	1058.671 $[M+Na]^+$	C53H93N7O13	1035.683	$C_{53}H_{93}N_7O_{13}$	Y	-	-	-
			Antibiotic BO 7: Antibiotic BO 7-2	1035.684	$C_{53}H_{93}N_7O_{13}$	-	Y	-	-
			Bacillus amyloliquefaciens surfactin 1	1035.684	$C_{53}H_{93}N_7O_{13}$	-	Y	-	-
			Bacillus pumilus KMM 1364 Lipodepsipeptides; KMM1364A	1035.684	$C_{53}H_{93}N_7O_{13}$	-	Y	-	-
			Bacillus pumilus KMM 1364 Lipodepsipeptides; KMM1364B	1035.684	$C_{53}H_{93}N_7O_{13}$	-	Y	-	-
			Bacircines; Bacircine 4	1035.684	$C_{53}H_{93}N_7O_{13}$	-	Y	-	-
			Bacircines; Bacircine 5	1035.684	$C_{53}H_{93}N_7O_{13}$	-	Y	-	-
			Daitocidin; Daitocidin A1	1035.684	$C_{53}H_{93}N_7O_{13}$	-	Y	-	-
			Gageopeptin A	1035.683	$C_{53}H_{93}N_7O_{13}$	Y	-	-	-
			Pumilacidin B	1035.683	$C_{53}H_{93}N_7O_{13}$	Y	-	-	-
			Surfactin	1035.683	$C_{53}H_{93}N_7O_{13}$	-	-	-	Y
			Surfactin; C15 surfactin	1035.684	$C_{53}H_{93}N_7O_{13}$	-	Y	-	-
			Surfactin; surfactin C1	1035.684	$C_{53}H_{93}N_7O_{13}$	-	Y	-	-
			Surfactin; surfactin C2	1035.684	$C_{53}H_{93}N_7O_{13}$	-	Y	-	-
			Surfactin C	1035.680	$C_{53}H_{93}N_7O_{14}$	-	-	Y	-
	1049.698 (18.6)	1050.705 $[M+H]^+$	Antibiotic BO 7: Antibiotic BO 7-3	1049.6988	$C_{54}H_{95}N_7O_{13}$	.	Y	.	.
			Bacillus pumilus KMM 1364 Lipodepsipeptides; KMM1364C	1049.6988	$C_{54}H_{95}N_7O_{13}$	.	Y	.	.
			Bacillus pumilus KMM 1364 Lipodepsipeptides; KMM1364D	1049.6988	$C_{54}H_{95}N_7O_{13}$	.	Y	.	.
			Daitocidin; Daitocidin B1	1049.6988	$C_{54}H_{95}N_7O_{13}$	.	Y	.	.
			Daitocidin; Pumilacidin F	1049.6988	$C_{54}H_{95}N_7O_{13}$	.	Y	.	.

			Daitocidin; Pumilacidin G	1049.6988	$C_{54}H_{95}N_7O_{13}$	.	Y	.	.
			Pumilacidin A	1049.6987	$C_{54}H_{95}N_7O_{13}$	Y	-	-	-
			Surfactin; ai-C16 surfactin	1049.6988	$C_{54}H_{95}N_7O_{13}$	.	Y	.	.
			Surfactin; surfactin D	1049.6988	$C_{54}H_{95}N_7O_{13}$	.	Y	.	.
	1063.713 (19.08)	1086.703 $[M+Na]^+$	*Bacillus pumilus* KMM 1364 Lipodepsipeptides; KMM1364E	1063.7144	$C_{55}H_{97}N_7O_{13}$	.	Y	.	.
			Daitocidin; Daitocidin A2	1063.7144	$C_{55}H_{97}N_7O_{13}$	.	Y	.	.
			Daitocidin; Daitocidin B2	1063.7144	$C_{55}H_{97}N_7O_{13}$	.	Y	.	.
			Pumilacidin D	1063.7144	$C_{55}H_{97}N_7O_{13}$	Y	-	-	-
			Pumilacidin E	1063.7144	$C_{55}H_{97}N_7O_{13}$	Y	-	-	-

Table S3. This table lists the samples that were analysed by 16S amplicon sequening, the primers were used per sample, and the accession numbers under which the amplicon datasets can be found in the ENA SRA database (project number: PRJEB31855).

Sample ID	Sample type	Forward primer components (5' to 3')					Reverse primer components (5' to 3')					Accession
		Illumina 5' Adapter	Barcode	Forward Primer Pad	Forward Primer Linker	515f forward primer	Illumina 5' Adapter	Barcode	Reverse Primer Pad	Reverse Primer Linker	806rB reverse primer	
NG_13961_H__leuco_gut_lib226555_5661	Internal (HL)	AATGATACGGCGACCACCGAGATCTACAC	CTACTATA	TATGGTAATT	GT	GTGYCAGCMGCCGCGGTAA	CAAGCAGAAGACGGCATACGAGAT	GTCGTAGT	AGTCAGTCAG	CC	GGACTACNVGGGTWTCTAAT	ERS3337754
NG_13961_H__leuco_skin_lib226556_5661	External (HL)		CTACTATA					TAGCAGAC				ERS3337755
NG_13961_S__vastus_gut_lib226557_5661	Internal (SV)		CGTTACTA					AACTCTCG				ERS3337756
NG_13961_S__vastus_skin_lib226558_5661	External (SV)		CGTTACTA					ACTATGTC				ERS3337757
NG_13961_Negative_control_product1_lib226684	Negative Control		ACGTCTCG					AGCTGCTA				ERS3337753

Table S4. Full-length sequence of 16S rRNA from SV 155.

Sample	Length (bp)	Next related type strain	Sequence
SV 155	1011	New genus of family Rhodobacteraceae (93.08% sequence similarity to *Paracoccus beibuensis*)	CTTCGGTTCTAGCGGCGGACGGGTGAGTAACGCGTGGGAACGTGCCCTTTGCTACGG AATAGCCCCGGGAAACTGGGAGTAATACCGTATGTGCCCCCTCAATCAAATTTCATTT GATTGAATTTTCAGTCTTATCGAATTCCGGATGGAATTTGATGGGGGGGAAAGATTT ATCGGCAAAGGATCGGCCCGCGTTGGATTAGGTAGTTGGTGGGGTAATGGCCTACCA AGCCGACGATCCATAGCTGGTTTGAGAGGATGATCAGCCACACTGGGACTGAGACA CGGCCCAGACTCCTACGGGAGGCAGCAGTGGGGAATCTTAGACAATGGGGGCAACC CTGATCTAGCCATGCCGCGTGAGTGATGAAGGCCTTAGGGTTGTAAAGCTCTTTCAGC TGGGAAGATAATGACGGTACCAGCAGAAGAAGCCCCGGCTAACTCCGTGCCAGCAG CCGCGGTAATACGGAGGGGGCTAGCGTTGTTCGGAATTACTGGGCGTAAAGCGCACG TAGGCGGATCGGAAAGTTGGGGGTGAAATCCCGGGGCTCAACCTCGGAACTGCCTTC AAAACTACTGGTCTGGAGTTCGAGAGAGGTGAGTGGAATTCCGAGTGTAGAGGTGA AATTCGTAGATATTCGGAGGAACACCAGTGGCGAAGGCGGCTCACTGGCTCGATACT GACGCTGAGGTGCGAAAGCGTGGGGAGCAAACAGGATTAGATACCCTGGTAGTCCA CGCCGTAAACGATGAATGCCAGTCGTCGGGTTGCATGCAATTCGGTGACACACCTAA CGGATTAAGCATTCCGCCTGGGGAGTACGGTCGCAAGATTAAAACTCAAAGGAATTG ACGGGGGCCCGCACAAGCGGTGGAGCATGTGGTTTAATTCGAAGCAACGCGCAGAA CCTTACCAACCCTTGACATCCCTGGACCGGCCCGGAGACGGGTCTTCCACTTCGGTGG CCAGGTGACAGGTGCTGCATGGCTGTCCTCAGCTCGTGTCCTGA
